# Neuropsychiatric Manifestations of Long COVID-19: A Narrative Review of Clinical Aspects and Therapeutic Approaches

**DOI:** 10.3390/life15030439

**Published:** 2025-03-11

**Authors:** Olga Adriana Caliman-Sturdza, Roxana Gheorghita, Andrei Lobiuc

**Affiliations:** 1 Faculty of Medicine and Biological Sciences, Stefan cel Mare University of Suceava, 720229 Suceava, Romania; olga.caliman-sturdza@usm.ro (O.A.C.-S.); andrei.lobiuc@usm.ro (A.L.); 2 Emergency Clinical Hospital Suceava, 720224 Suceava, Romania

**Keywords:** long COVID-19, quality of life, neurological symptoms, pathophysiological mechanisms, risk factors

## Abstract

The COVID-19 (C-19) pandemic has highlighted the significance of understanding the long-term effects of this disease on the quality of life of those infected. Long COVID-19 (L-C19) presents as persistent symptoms that continue beyond the main illness period, usually lasting weeks to years. One of the lesser-known but significant aspects of L-C19 is its impact on neuropsychiatric manifestations, which can have a profound effect on an individual’s quality of life. Research shows that L-C19 creates neuropsychiatric issues such as mental fog, emotional problems, and brain disease symptoms, along with sleep changes, extreme fatigue, severe head pain, tremors with seizures, and pain in nerves. People with cognitive problems plus fatigue and mood disorders experience great difficulty handling everyday activities, personal hygiene, and social interactions. Neuropsychiatric symptoms make people withdraw from social activity and hurt relationships, thus causing feelings of loneliness. The unpredictable state of L-C19 generates heavy psychological pressure through emotional suffering, including depression and anxiety. Neuropsychiatric changes such as cognitive impairment, fatigue, and mood swings make it hard for people to work or study effectively, which decreases their output at school or work and lowers their job contentment. The purpose of this narrative review is to summarize the clinical data present in the literature regarding the neuropsychiatric manifestations of L-C19, to identify current methods of diagnosis and treatment that lead to correct management of the condition, and to highlight the impact of these manifestations on patients’ quality of life.

## 1. Introduction

COVID-19 (C-19) represents an infectious disease, with SARS-CoV-2 virus as the infectious agent, encountered for the first time in late 2019, thereafter quickly spreading worldwide and resulting in a pandemic. Through its main impact on the respiratory system, C-19 induces symptoms that extend from light fever and cough to intense pneumonia and acute respiratory distress syndrome (ARDS) [1]. The virus primarily travels through respiratory droplets and requires direct contact; yet, asymptomatic and pre-symptomatic individuals also help spread infection. Most C-19 cases recover effectively, but elderly individuals, together with people who have multiple health conditions, experience serious health complications and higher death rates. Following the acute stage of illness, C-19 leads to increased risks of cardiovascular issues and neurological conditions, together with neuropsychiatric disorders. C-19 variants, including Alpha, Delta, and Omicron, have turned the tide on how easily the virus spreads, how effectively it evades immune response, and how severe the disease is in affected cases. The focus of pandemic control has aligned with three main strategies, namely, vaccine development and distribution, antiviral drug treatments, and public hygiene safety rules such as mask requirements and social/physical space regulations. C-19 causes extensive health problems worldwide, resulting in respiratory complications and systemic reactions, together with noticeable neuropsychiatric complications. At the beginning of the pandemic, researchers explored investigative therapies, including antiviral drugs and immune system-oriented treatments, to reduce the impact of the disease [2]. Ongoing investigations have become vital to knowing all virus modification aspects and creating effective medical solutions because C-19 is transforming with time. Long COVID-19 (L-C19), also known as post-COVID syndrome or post-acute sequelae of SARS-CoV-2 infection (PASC), refers to a collection of symptoms that persist or emerge weeks to months after an acute C-19 infection is resolved [3,4,5]. While many individuals recover from C-19 within a few weeks, a significant subset of patients experience ongoing health issues, with neuropsychiatric manifestations being among the most common and disabling ones [6]. The neuropsychiatric manifestations of L-C19 encompass a wide range of symptoms affecting mental health and cognitive function, and they can significantly impact the quality of life of affected individuals. These symptoms often involve cognitive impairments, mood disorders, and other psychological distress that persist long after the initial viral infection [7]. Notably, many individuals with L-C19 experience brain fog, anxiety, depression, sleep disturbances, and memory issues, making it difficult for them to resume normal daily activities, return to work, or maintain social relationships [8]. The SARS-CoV-2 virus has developed several variants, such as Delta and Omicron, which have modified both the symptomatic expressions and medical severity of C-19. The Delta variant emerged late in 2020; it transmitted faster between people and made the illness more severe than previous variants. Research shows that the Omicron variant spread rapidly in late 2021, generally causing less severe acute illness, although it transmitted better than the other variants [7,8]. Scientists are currently investigating Omicron’s effect on L-C19 rates, including neuropsychiatric syndromes. The severity of a patient’s first C-19 infection remains the main factor that determines their risk of neuropsychiatric conditions over the long term [3,6].

Publications in the field have presented the most important neurological manifestations and complications due to L-C19 [9,10,11,12], and some of them indicated clinical management [13,14,15]. The purpose of this review is to highlight the neurological and psychiatric clinical manifestations that classify a patient as having post-C-19 sequelae, explore current diagnostic methods and appropriate management of these neuropsychiatric manifestations, examine available treatment options, and, last but not least, assess the impact of these changes on quality of life. It is necessary that clinicians have better knowledge and understanding of the clinical manifestations and monitoring possibilities of patients with L-C19 in order to make all the necessary efforts for a full recovery.

## 2. Methods

This paper is based on a critical literature review using the PubMed, Google Academic, and EMBASE databases, which were used to access articles published before 31 December 2024 using keywords (title and abstract) such as “long COVID-19”, “post-Acute COVID-19 syndrome”, “long COVID-19 neuropsychiatric manifestation”, “neurological manifestation of Long COVID-19”, “psychiatric manifestation of long COVID-19”, “cognitive disorders”, “headache”, “brain fog”, “sleep disturbance”, “anxiety and depression in Long COVID-19”, and “quality of life.” In this analysis, we included articles such as systematic reviews, narrative reviews, evidence-based clinical guidelines, randomized clinical trials, and observational studies. To obtain additional data for this narrative review, we also checked the bibliography of relevant articles identified through a manual search. Additionally, we sought the latest available data in the literature on the neuropsychiatric consequences of L-C19. As inclusion criteria, we used only peer-reviewed articles written in English and published in Web of Science databases. We excluded abstracts from congresses or studies that did not undergo the peer review process. The selection of the articles that were the basis of this review was made with the consent of all authors.

## 3. Results and Discussion

### 3.1. Prevalence and Impact

**Figure 1:**
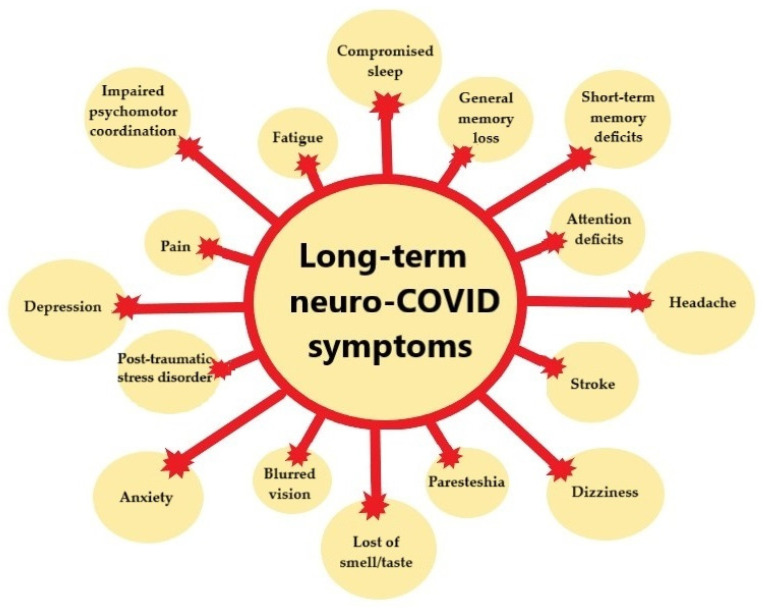
Most common L-C19 neurological symptoms.

The incidence of neuropsychiatric manifestations of L-C19 varies widely across studies, with reported rates influenced by factors such as study population, the severity of acute C-19 infection, and the methods used for diagnosis. Neuropsychiatric symptoms in L-C19 can range from mild cognitive issues, such as brain fog, to severe mental health disorders, including anxiety, depression, and post-traumatic stress disorder (PTSD) [16] (Figure 1).

Cognitive dysfunction, or “brain fog”, is one of the most commonly reported neuropsychiatric symptoms of L-C19. Estimates suggest that 40% to 60% of individuals with L-C19 experience cognitive difficulties, including memory issues, difficulty concentrating, and executive functioning problems [17]. A study published in JAMA Neurology reported that approximately 30% to 50% of patients with L-C19 experienced some degree of cognitive dysfunction [18]. Depression and anxiety are highly prevalent among individuals with L-C19, with estimates of up to 30% to 40% of patients experiencing these disorders [19]. Recent research found that 22% to 28% of L-C19 patients reported symptoms of depression, while 23% to 30% reported anxiety [20]. These figures suggest a significantly higher prevalence of mental health conditions in L-C19 compared to the general population or pre-pandemic rates.

Sleep disorders, including insomnia and non-restorative sleep, are also common. Approximately 25% to 40% of individuals with L-C19 report sleep disturbances, contributing to fatigue and mood instability [21]. Poor sleep quality is associated with worsened cognitive symptoms and mental health outcomes.

PTSD has been reported in a subset of L-C19 patients, particularly those who experience severe acute C-19 symptoms or prolonged hospitalization. Another high-profile study found that approximately 20% to 30% of patients with L-C19 reported PTSD-like symptoms, especially those who had severe illness or were in intensive care units (ICUs) [21]. PTSD symptoms may also result from the trauma of illness, social isolation, and the prolonged uncertainty surrounding recovery [22]. Psychosis and more severe psychiatric conditions, while less common, have been reported in a smaller percentage of patients. Estimates suggest that 1% to 3% of L-C19 patients may experience psychotic symptoms, such as delusions or hallucinations [23]. Fatigue and mood swings are also common, with around 30% to 50% of individuals reporting significant levels of fatigue that contribute to emotional and cognitive issues [24,25].

### 3.2. Pathophysiology

The pathophysiology of neuropsychiatric manifestations of L-C19 is complex and not yet fully understood, but it is believed to involve several interrelated mechanisms [26].

#### 3.2.1. Immune System-Related Mechanisms

**Figure 2:**
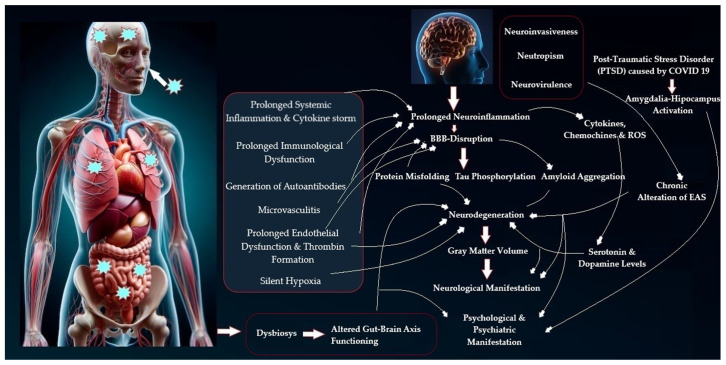
Pathophysiological mechanisms implicated in neuropsychiatric L-C19 manifestation (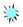
—C19)The mechanisms underlying the neuropsychiatric manifestations of L-C19 are complex and interact with each other: 1. Persistent systemic inflammation and cytokine storms: In L-C19 patients, SARS-CoV-2 creates immunological irregularities, which trigger neuroinflammatory responses by activating microglia cells and driving the development of brain white matter abnormalities and microvascular damage. 2. Neuroinvasiveness, neurotropism, and neurovirulence: Through the ACE2 receptor, SARS-CoV-2 shows inherent neuroinvasive capabilities, enabling it to attack neuronal cells and set off cellular death in nearby tissue. 3. Blood–brain barrier (BBB) disruption: Brain injury from SARS-CoV-2 causes integrity damage to the blood–brain barrier, which results in higher permeability and allows blood component leakage, along with immune cell transmission throughout brain parenchyma. 4. Prolonged neuroinflammation: The brain experiences a persistent inflammatory state due to cytokines and chemokines that persistently activate brain tissue. 5. Microvasculitis: Brain inflammation persists in C-19 patients, leading to microvascular damage throughout the brain. 6. Prolonged endothelial dysfunction, platelet activation, and enhanced thrombin generation: These factors, in addition to others, may disrupt brain regions’ functioning in patients experiencing neuro-L-C19. 7. Silent hypoxia contributes to brain damage while encouraging cytokine storms through inflammatory mediators, which cause severe damage to the endothelial system. 8. Dysregulated levels of neurotransmitters like serotonin and dopamine: Brain dysfunction could lead to neurological and psychological symptoms during L-C19 development. 9. Amyloid aggregation: Numerous viral proteins demonstrate the capacity to produce amyloid proteins. 10. Neuronal death: The two biological mechanisms that explain neuronal damage in L-C19 are astrogliosis and microgliosis, both induced by SARS-CoV-2. 11. Tau phosphorylation could contribute to memory problems. 11. Changes in grey matter volume: The latest scientific research indicates that C-19 infections transform cortical grey matter volume in human bodies. 12. Prolonged immunological dysfunction: This condition appears regularly in infected patients while causing persistent neurological and psychiatric symptoms of L-C19. 13. Altered gut–brain axis functioning: Inflammatory processes that continue over time produce shifts in both gut microbiome composition and neural–immune system interactions. L-C19 patients show both poor mental test results and gastrointestinal symptoms, which have been linked to abnormal sleep patterns. 14. Chronic alteration of the extended autonomic system (EAS), including the neuroendocrine and neuroimmune systems: Continued activation of the extended autonomic system in C-19 patients could potentially direct regulatory systems toward dysfunction. Epidemiological studies have shown that L-C19 patients exhibit signs of reduced cortisol levels, which is associated with depression. 15. Post-traumatic stress disorder and increased amygdala and hippocampal activity: Imaging studies have shown higher levels of amygdala activation among patients with L-C19, while hippocampal region activity shows a solid connection to the depressive symptoms seen in this group.

It is hypothesized that there is an interaction between multiple potential neuroimmune mechanisms specific to SARS-CoV-2 infections, including persistent inflammation, autoimmunity, direct virus-mediated cytotoxicity, hypercoagulation, mitochondrial failure, dysbiosis, and a reactivation of other persistent viral infections [27]. One of the most prominent mechanisms in L-C19 is chronic activation of the immune system. The virus can trigger persistent inflammation in the brain and nervous system, even after the acute infection resolves [27,28]. This immune response is thought to contribute to cognitive and psychiatric symptoms. C-19 is associated with a heightened inflammatory response, often referred to as a cytokine storm, where the body releases a large number of pro-inflammatory cytokines, for example, interleukin 6 (IL-6) and tumor necrosis factor (TNF-α) [29,30]. These cytokines can cross the blood–brain barrier and influence brain function, contributing to symptoms such as fatigue, cognitive dysfunction, mood disturbances, and sleep problems [31]. Glial cells, which support neurons in the brain, can become activated during an immune response. Chronic activation of these cells may contribute to neuroinflammation and result in the persistence of neuropsychiatric symptoms [32]. Microglia are immune cells in the brain that act as the first line of defense. Chronic activation of microglia can lead to neuroinflammation, which may be responsible for the cognitive issues and mood disorders in L-C19 patients [33,34,35]. SARS-CoV-2 may directly invade the central nervous system (CNS) through multiple mechanisms, including the olfactory nerve, which is responsible for smell [36,37]. Some studies have reported that the SARS-CoV-2 virus enters the central nervous system through the olfactory nerve, the trigeminal nerve, or the nerve endings of the vagus nerve through retrograde transport mechanisms. There is evidence that the virus may be able to enter the brain by binding to angiotensin-converting enzyme 2 (ACE2) receptors, which are found on neurons and glial cells [37,38,39]. Once inside the brain, the virus can potentially damage brain tissue and trigger inflammation, contributing to symptoms like brain fog, headaches, and cognitive decline [40]. The virus is also thought to affect cranial nerves, particularly the olfactory nerve, which could explain the loss of smell (anosmia), as often seen in C-19 patients [37,40]. This sensory disturbance could also be related to broader neuropsychiatric dysfunction (Figure 2).

There is growing evidence that C-19 may trigger an autoimmune response, where the body’s immune system mistakenly attacks its own tissues, including the brain. This phenomenon, called molecular mimicry, occurs when the immune system targets neural tissue because it resembles the virus [41]. This may contribute to symptoms such as cognitive dysfunction, mood changes, and other neurological manifestations. Even after the viral infection clears, the immune system can remain in a state of heightened activity [42]. This persistent immune dysregulation may cause prolonged inflammation in the CNS, leading to the ongoing neuropsychiatric symptoms of L-C19 [43,44]. Another mechanism is that SARS-CoV-2 infection can lead to dysfunction of the blood–brain barrier, making it more permeable [45,46]. This allows pro-inflammatory molecules, immune cells, and even viral particles to enter the brain more easily, leading to inflammation and contributing to cognitive and mood disturbances [47].

#### 3.2.2. Vascular System-Related Mechanisms

Additionally, C-19 is associated with increased blood clotting and vascular injury, which could disrupt normal blood flow to the brain [48]. This vascular damage may result in ischemia or microvascular changes, leading to cognitive decline and other neuropsychiatric symptoms [49]. During the acute phase of C-19, some patients experience severe hypoxia, which is a lack of adequate oxygen levels in the blood. Prolonged hypoxia can damage brain tissue, leading to cognitive impairment, mood changes, and even psychosis in some cases [50,51]. Even after recovery from the acute infection, individuals who experienced significant oxygen deprivation during their illness may continue to experience neurological symptoms due to the long-term effects of hypoxia on brain function [52]. The virus and its effects on the immune system may alter brain chemistry by impacting neurotransmitters such as serotonin, dopamine, and glutamate. Studies suggest that L-C19 is associated with low serotonin levels, possibly due to persistent inflammation, gut microbiome changes, and disrupted serotonin metabolism. There is evidence that L-C19 can lead to dopaminergic dysfunction, likely due to neuroinflammation and oxidative stress [53].

These changes can contribute to psychiatric symptoms like anxiety, depression, and cognitive dysfunction [54]. The alteration of serotonin levels, a neurotransmitter involved in mood regulation, is often associated with depression and anxiety [55]. In L-C19, the dysregulation of serotonin pathways may contribute to the persistent mood disorders seen in many patients. Changes in dopamine pathways may affect motivation, pleasure, and mood, contributing to fatigue, apathy, and a lack of interest in activities. Chronic stress triggered by the illness and its consequences can lead to the dysregulation of the hypothalamic–pituitary–adrenal (HPA) axis, which is responsible for managing the body’s stress response [56]. Overactivation of the stress system can lead to mood disorders such as depression, anxiety, and irritability. Prolonged stress or immune activation may cause an imbalance in cortisol levels.

#### 3.2.3. Hormone- and Mitochondria-Mediated Mechanisms

High or low cortisol levels can negatively affect mood, cognition, and overall mental health [29,57]. Emerging research indicates that dysfunction of the HPA axis may play a significant role in the pathophysiology of L-C19. The HPA axis is crucial for stress response and immune regulation, and its disruption can lead to a range of persistent symptoms observed in L-C19 patients [49,50]. Several mechanisms have been proposed to explain how SARS-CoV-2 infection might impair the HPA axis. SARS-CoV-2 can infect tissues expressing ACE2 receptors, which are present in the hypothalamus, pituitary, and adrenal glands. This direct invasion may lead to inflammation and damage within these structures, potentially resulting in conditions like hypophysis or adrenalitis [58]. The infection may trigger the production of antibodies against adrenocorticotropic hormone (ACTH), leading to reduced cortisol production and adrenal insufficiency [59]. Severe C-19 can lead to critical illness-related corticosteroid insufficiency (CIRCI), where the body’s cortisol response is inadequate for the level of stress, potentially due to impaired adrenal function or disrupted signaling within the HPA axis [58]. Persistent HPA axis dysfunction may manifest as chronic fatigue, muscle weakness, cognitive disturbances (brain fog), and other symptoms commonly reported in L-C19 cases. A study highlighted that 13% of patients exhibited adrenal insufficiency 12 months post-C-19 infection, with all cases being of hypothalamic–pituitary origin. Notably, some patients developed adrenal insufficiency after recovery from the acute phase, suggesting a delayed onset in certain individuals [60].

L-C19 can lead to dysautonomia, which refers to the malfunction of the autonomic nervous system (ANS), which is responsible for controlling automatic functions such as heart rate, blood pressure, and digestion. This dysregulation can cause symptoms like postural orthostatic tachycardia syndrome (POTS), dizziness, and fatigue, which often accompany neuropsychiatric issues like anxiety and cognitive impairment [61,62]. These fluctuations can affect blood flow to the brain and may contribute to feelings of lightheadedness, difficulty concentrating, and emotional instability [63]. The SARS-CoV-2 virus may interfere with mitochondrial function, which is essential for cellular energy production. Dysfunction in mitochondria can lead to fatigue, cognitive dysfunction, and mood disturbances as the brain is highly sensitive to changes in energy metabolism [64]. The experience of ongoing illness and the uncertainty about recovery can contribute significantly to psychiatric symptoms such as anxiety, depression, and PTSD [65]. The stress of dealing with persistent symptoms, financial difficulties, or the emotional toll of being ill for a prolonged period may exacerbate existing mental health conditions or create new ones. Prolonged periods of isolation during the acute phase of C-19, especially during lockdowns or quarantine, may increase feelings of loneliness, depression, and anxiety. These social and psychological stressors can contribute to L-C19-related neuropsychiatric manifestations [64,66].

## 4. Risk Factors for Neuropsychiatric Manifestations of L-C19

The neuropsychiatric manifestations of L-C19 can be influenced by various risk factors. These factors may be biological, psychological, or environmental, and understanding them can help identify individuals who may be at higher risk of developing these symptoms after C-19 infection [67].

### 4.1. Hospital Stay-Induced Risks

Individuals who were hospitalized, especially those who required ICU care or ventilatory support, are at a higher risk of developing neuropsychiatric symptoms in the long term [68]. The more severe the acute infection, the more likely there will be lasting effects on the brain and mental health. Severe hypoxia and the need for intubation can cause lasting damage to brain tissue and are associated with a higher risk of cognitive impairment and mood disorders. The longer the acute illness lasts, the more likely it is that neuropsychiatric symptoms will emerge during recovery due to the prolonged stress on the body and brain [69].

### 4.2. Hormone-Related Risks

Individuals with a history of depression, anxiety, bipolar disorder, or PTSD are at greater risk of developing or exacerbating neuropsychiatric symptoms after C-19 [65]. The stress of illness, social isolation, and post-infection challenges can trigger or worsen these conditions [70]. Studies suggest that women are at a higher risk of experiencing neuropsychiatric symptoms during L-C19 [71]. Women are more likely to report cognitive symptoms like brain fog, fatigue, and mood disorders such as anxiety and depression [72]. Hormonal differences and differences in immune response may contribute to this increased vulnerability. Estrogen and progesterone levels, which are higher in women, may influence immune responses and brain function. Some research suggests that hormonal fluctuations may contribute to the increased vulnerability of women to neuropsychiatric symptoms, such as anxiety, depression, and cognitive dysfunction, particularly after infection [73,74].

### 4.3. Aging and Chronic Diseases as Risk Factors

Elderly individuals are more likely to experience persistent neuropsychiatric symptoms, including cognitive decline and depression, during L-C19 [75]. Aging is associated with increased vulnerability to brain inflammation and damage, which may make recovery from the neurological effects of C-19 more challenging. Although older adults are more affected, there is evidence that younger individuals, especially those with severe illness, can experience significant neuropsychiatric manifestations like anxiety, fatigue, and memory problems [76]. However, the full impact on the younger population remains an area of active research. People with chronic health conditions such as diabetes, hypertension, cardiovascular diseases, chronic respiratory conditions (e.g., asthma), and obesity may be at higher risk of developing neuropsychiatric manifestations [77,78]. These conditions often make recovery from C-19 more difficult and can increase inflammation and vulnerability to mood disorders [79]. Individuals with autoimmune diseases (e.g., rheumatoid arthritis and lupus) may experience a more intense immune response to C-19, increasing the risk of neuropsychiatric symptoms due to immune system dysregulation [80,81]. People with a history of stroke, multiple sclerosis, epilepsy, or other neurological disorders may experience worsened cognitive and psychiatric symptoms following C-19 infection due to the added strain on the nervous system [82]. Individuals who had some degree of cognitive decline or mild cognitive impairment (MCI) before infection are at a higher risk of developing more severe cognitive issues (e.g., brain fog) during L-C19 [83]. Individuals with an overactive immune response during the acute phase of C-19, such as those who experience a cytokine storm or severe inflammation, may be more likely to develop persistent neuropsychiatric symptoms [84]. Dysregulated immune responses can trigger chronic inflammation in the brain, which is thought to contribute to mood disorders, cognitive dysfunction, and other neurological symptoms [85,86]. The lockdowns, quarantine measures, and fear of infection associated with the C-19 pandemic have led to significant social isolation. People who experience extended isolation or lack of social support are at greater risk of developing depression, anxiety, and other psychiatric conditions [87]. The emotional toll of the pandemic, such as dealing with loss, uncertainty, financial strain, or severe illness, can be a trigger for post-traumatic stress disorder (PTSD), depression, and anxiety. The stress of navigating L-C19 symptoms can also exacerbate pre-existing mental health conditions [88]. People with existing sleep apnea or respiratory problems may be more vulnerable to the neuropsychiatric effects of C-19 due to the impact of poor sleep on cognitive function and emotional regulation [89,90].

### 4.4. Genetics- and Lifestyle-Related Risks

There may be genetic factors that predispose individuals to experience more severe neuropsychiatric symptoms following C-19. Variants in genes related to immune function, neurotransmitter regulation, or stress response pathways might make certain individuals more vulnerable to psychiatric symptoms after infection. Chronic illness, stress, and inflammation can lead to epigenetic changes that affect gene expression. These modifications might influence susceptibility to neuropsychiatric symptoms, and some individuals may experience more significant mental health challenges due to these genetic and environmental interactions [91,92,93]. Reduced physical activity, either due to illness or the fear that exercise may exacerbate symptoms, can lead to a decline in mental health. Physical exercise is known to improve mood, reduce stress, and improve cognitive function [94,95]. The use of unhealthy coping mechanisms, such as alcohol consumption or substance abuse, can worsen psychiatric symptoms during L-C19 recovery [96]. Individuals who have a history of other post-viral conditions, such as chronic fatigue syndrome or mononucleosis (Epstein–Barr virus infection), may be more predisposed to experiencing L-C19 and its associated neuropsychiatric symptoms due to similar mechanisms of immune dysregulation, inflammation, and fatigue. In contrast, subjects with serologic evidence of prior cytomegalovirus infection are less likely to develop chronic neurocognitive manifestations of SARS-CoV-2 infection [29,97].

## 5. Neuropsychiatric Manifestations of L-C19

This narrative review took into account 271 review articles, meta-analyses, research articles, including articles that focused on LC-19 and especially the neuropsychiatric manifestations of LC-19, published in the 2020–2024 period in English. All the selected articles were reviewed by the three authors of this study.

### 5.1. Neurological Manifestations of L-C19

Neurological manifestations of L-C19 are a diverse set of symptoms that affect the central and peripheral nervous systems. These symptoms can persist long after the acute phase of C-19 and can vary in severity. Neurological complications are common in individuals with L-C19 and can significantly impact daily functioning and quality of life. The main neurological manifestations associated with L-C19 are cognitive impairments (cognitive dysfunction or brain fog), memory problems (difficulty recalling recent events, conversations, or appointments), concentration difficulties (trouble focusing on tasks, following conversations, or completing complex tasks), slowed thinking, and impaired executive functioning (difficulty with organizing, planning, and decision making) [98]. These cognitive issues are often described as brain fog, and they are one of the most commonly reported neurological symptoms in L-C19 patients [99]. Another symptom is chronic or persistent headaches, which can range from mild to severe and may resemble tension headaches or migraines [100,101,102]. The headaches often occur alongside other symptoms, such as fatigue and dizziness. For individuals with a history of migraines, L-C19 may lead to more frequent or intense episodes. Peripheral neuropathy can lead to sensations of tingling, numbness, or a “pins and needles” feeling, often in the hands and feet [103]. L-C19 can cause muscle weakness or atrophy, contributing to difficulty with movement. Chronic pain in various parts of the body, including muscles and joints, may be present [104]. Patients with L-C19 may experience vertigo (sensation of spinning or loss of balance) and dizziness, which may be triggered by movement or standing up [103]. POTS is a condition characterized by an abnormal increase in heart rate when standing up, often accompanied by dizziness, lightheadedness, and fainting. It is thought to be related to dysautonomia and is a common neurological manifestation in L-C19 [105]. There have been reports of patients with L-C19 experiencing seizures, although this is relatively rare [106]. The exact cause of seizures in L-C19 patients is not well understood, but it may involve brain inflammation, structural changes, or disruption of electrical activity in the brain. Some individuals experience tremors or involuntary shaking, which can affect the hands, legs, or other parts of the body, and may have problems with coordination (ataxia), bradykinesia, or myoclonus. Tremors in L-C19 may be related to nervous system involvement or medication side effects [51,107]. Encephalopathy refers to any diffuse disease of the brain that results in impaired brain function. This can manifest as confusion, memory loss, difficulty concentrating, and changes in behavior. In some cases, individuals with L-C19 report persistent feelings of confusion or disorientation, which may be linked to encephalopathy [108]. While rare, there have been reports of patients experiencing strokes during or after a C-19 infection. C-19 may increase the risk of blood clots, leading to stroke in some patients, particularly in those with pre-existing cardiovascular risk factors [109,110]. Damage or inflammation to cranial nerves can cause a variety of symptoms, such as anosmia or ageusia, which is particularly associated with the early stages of C-19 but may persist in L-C19. Some patients may present facial weakness or paralysis, similar to Bell’s palsy, or vision problems, such as double vision or difficulty focusing, often related to the nerves controlling eye movement [111,112]. Insomnia, non-restorative sleep, or frequent awakenings can be partly neurological in nature, such as disruptions in brain activity during sleep. Sleep disorders are often associated with cognitive dysfunction and can contribute to other neurological symptoms [113]. These manifestations have been reported by many patients, as can be observed in Table 1.

Many neurological manifestations of L-C19 overlap with psychiatric symptoms, such as anxiety and depression, which are often seen alongside cognitive impairments. These psychiatric symptoms can exacerbate neurological symptoms, creating a complex interaction between mental and physical health [115]. Muscle weakness and atrophy may result from prolonged bed rest, deconditioning, or inflammation. Some individuals with L-C19 report ongoing muscle aches, stiffness, or general fatigue, which impair movement [122]. Dysautonomia refers to disturbances in the autonomic nervous system, which controls involuntary functions like heart rate, blood pressure, digestion, and temperature regulation. Symptoms can include fluctuating blood pressure, difficulty regulating body temperature, and abnormal sweating, in addition to POTS [123].

### 5.2. Psychiatric Manifestations of L-C19

Psychiatric manifestations of L-C19 are common and can significantly affect individuals’ mental health and overall well-being. These symptoms may persist long after the acute phase of C-19 is resolved and can impact mood, behavior, cognition, and social functioning [54]. Generalized anxiety disorder (GAD) consists of persistent and excessive worry about a range of issues, often with physical symptoms such as restlessness, fatigue, and difficulty concentrating [124]. Panic disorders are recurrent, with unexpected panic attacks that involve intense fear or discomfort, often accompanied by physical symptoms like heart palpitations, sweating, and shortness of breath [125,126]. Patients may experience health anxiety, an exaggerated fear of having or developing severe health conditions, and social anxiety, an increased fear or anxiety about social interactions, especially in those who have been isolated during illness or lockdown periods [127]. Major depressive disorder (MDD) can be expressed by persistent feelings of sadness, hopelessness, and a lack of interest in activities once enjoyed. Symptoms can include low mood, irritability, feelings of emptiness, fatigue, sleep disturbances, changes in appetite, and reduced ability to concentrate or make decisions [128]. A subtype of depression may arise after viral infection, often triggered by the stress of the illness, physical symptoms, or the uncertainty surrounding recovery. Some individuals with L-C19 reported rapid shifts between extreme emotional highs (euphoria or irritability) and lows (depression or despair) [129]. These mood fluctuations may be due to brain inflammation, hormonal changes, or the impact of prolonged illness [130]. The ongoing physical and mental strain of L-C19 may lead to emotional instability, frustration, or a quick temper. The experience of severe illness, hospitalization, and the fear of death associated with C-19 may contribute to PTSD in some individuals [131,132]. Symptoms of PTSD may include intrusive thoughts or flashbacks, nightmares, hypervigilance, and avoidance of reminders related to the traumatic experience (such as avoiding healthcare settings or discussions about C-19). The stress of dealing with the persistent symptoms of L-C19 and uncertainty about recovery can exacerbate or lead to PTSD-like symptoms [133,134]. Cognitive dysfunction (brain fog) is often seen in tandem with psychiatric symptoms like anxiety and depression; brain fog can contribute to difficulty in concentrating, processing information, and remembering tasks [134]. The frustration of cognitive challenges can worsen mental health conditions. Psychiatric symptoms, such as anxiety and depression, often coexist with sleep disturbances like insomnia or non-restorative sleep. Poor sleep can, in turn, aggravate mood and cognitive issues [135]. Fatigue is a hallmark of L-C19 and can often lead to a sense of apathy, a lack of motivation, and withdrawal from activities. Severe fatigue can contribute to feelings of being overwhelmed or hopeless, which may lead to a loss of interest in socializing or engaging in previously enjoyed activities [136]. Some individuals may experience confusion, disorientation, or difficulty thinking clearly, which may present as a form of delirium. Although less common, some individuals with L-C19 have reported experiencing hallucinations or delusions, often in the context of severe illness or delirium [137]. In some cases, individuals with L-C19 may develop new or intensified symptoms of obsessive–compulsive disorder (OCD), including intrusive thoughts and compulsive behaviors (e.g., excessive hand washing or sanitizing). Health-related obsessive thoughts, fears of reinfection, or compulsive checking of symptoms may manifest in individuals recovering from C-19 [138,139]. Many individuals with L-C19 experience social withdrawal, driven by both physical fatigue and psychological factors such as anxiety, depression, or shame related to their symptoms. The inability to participate in social activities or communicate effectively due to cognitive and emotional challenges can reduce social interactions, leading to further isolation and loneliness [140]. Long-term illness and chronic symptoms can cause individuals to struggle with their sense of self and identity. This may be related to a loss of work function, inability to engage in hobbies, or frustration with ongoing symptoms. Dealing with illness, cognitive challenges, and mood instability may contribute to feelings of inadequacy or guilt [141]. The combination of physical symptoms (e.g., chronic pain and fatigue) and mental strain can lead to increased irritability or agitation, sometimes leading to frustration with medical systems or feelings of helplessness [142].

## 6. Diagnosing Neuropsychiatric Manifestations of L-C19

Diagnosing neuropsychiatric manifestations of L-C19 involves a multi-faceted approach as these symptoms can range from cognitive impairments to mood disorders [54]. The diagnosis typically requires a combination of clinical evaluation, self-reported symptom questionnaires, neuropsychological testing, and exclusion of other potential causes (Table 2).

Since L-C19 is a complex and emerging condition, accurate diagnosis is important to guide treatment and support for affected individuals [143]. The first step in diagnosis is a thorough clinical interview, which includes symptom onset, duration, and severity. Typically, the symptoms occur four weeks or more after the acute phase of C-19 and last for at least two months [3,4]. The clinician should also assess pre-existing mental health conditions or any cognitive issues prior to the infection that might influence symptom presentation [144]. Several standardized questionnaires and scales can help screen for the most common neuropsychiatric symptoms seen in L-C19 (Table 3).

Neuropsychological tests are performed to assess cognitive domains like attention, memory, executive functioning, processing speed, and language. These tests are typically administered by a neuropsychologist and may involve paper-and-pencil tasks, computerized assessments, or structured interviews [151]. Neuropsychological assessment helps distinguish between cognitive impairments due to L-C19 and other potential causes, such as underlying neurodegenerative diseases (e.g., Alzheimer’s) or depression-related cognitive changes [157]. While neuropsychiatric symptoms are a hallmark of L-C19, it is important to rule out other medical, psychiatric, or neurological conditions that may be contributing to these symptoms. Laboratory tests may be used to determine other causes of neuropsychiatric symptoms, including thyroid function tests such as Thyroid-Stimulating Hormone (TSH) and Thyroxine (T4) levels and vitamin levels (e.g., B12 and D) [158,159]. Deficiencies in these vitamins can contribute to fatigue, cognitive dysfunction, and mood disorders. A complete blood count (CBC) can rule out anemia, infections, or other hematologic issues. Liver and kidney function tests may be performed to assess for any organ dysfunction that could cause cognitive impairment [160,161,162]. To exclude autoimmune disorders that could mimic neuropsychiatric symptoms (e.g., rheumatoid factor and anti-nuclear antibodies), it is necessary to perform autoimmune markers [161]. Magnetic resonance imaging (MRI) or computed tomography (CT) scans of the brain can be ordered if a neurological cause is suspected or if the patient presents with focal neurological symptoms (e.g., persistent headaches, motor weakness, or seizures) [160]. However, imaging is not typically required to diagnose L-C19 unless there is concern about other underlying neurological conditions (e.g., stroke or multiple sclerosis). If seizures or other unusual neurological symptoms are present, an electroencephalogram (EEG) can be used to assess brain activity [163]. Some patients may meet the criteria for chronic fatigue syndrome (CFS), which shares many similar symptoms with L-C19 [164]. In such cases, it may be important to assess for fatigue, non-restorative sleep, and muscle or joint pain, which can overlap with both neuropsychiatric and physical symptoms of L-C19. Given the complexity of L-C19, a multidisciplinary approach to diagnosis and management is often recommended. This may include neurologists for the assessment of brain function and cognitive testing, psychiatrists or psychologists for mental health evaluation and management, sleep specialists for sleep disorders, and rehabilitation specialists for cognitive rehabilitation and physical therapy to manage symptoms of fatigue or mobility issues. L-C19 symptoms often overlap with other psychiatric conditions, making diagnosis challenging. For example, fatigue, brain fog, and depression are also common in chronic fatigue syndrome and major depressive disorder. Neuropsychiatric symptoms can be highly variable, and some patients may experience fluctuating or mild symptoms that are difficult to quantify or diagnose. As of now, there are no specific biomarkers for diagnosing the neuropsychiatric manifestations of L-C19. Diagnosis is largely based on clinical criteria and symptom tracking.

## 7. Treatment of Neuropsychiatric Manifestations of L-C19

The treatment of neuropsychiatric manifestations of L-C19 is complex and multidisciplinary, addressing a range of symptoms such as cognitive dysfunction (e.g., brain fog), mood disorders (e.g., anxiety and depression), sleep disturbances [165], and fatigue (Table 4).

Management strategies should be tailored to the individual based on the severity of symptoms, comorbid conditions, and the patient’s overall health [187]. Cognitive behavioral therapy (CBT) can be beneficial for patients experiencing cognitive symptoms, particularly those with anxiety or depression, and this may contribute to brain fog [171]. CBT is the gold standard for managing both depression and anxiety. It helps patients develop coping strategies and address negative thought patterns associated with L-C19. Cognitive training programs may involve specific exercises designed to improve memory, attention, and problem-solving skills [166]. Cognitive exercises or computerized programs such as brain training apps may help improve executive functioning. Using external tools such as reminders, planners, or apps to help manage memory and cognitive tasks can reduce the impact of brain fog on daily functioning. Adequate rest and good sleep hygiene are crucial for cognitive recovery [188,189,190]. Poor sleep exacerbates cognitive dysfunction. Some studies have investigated the mobile application ReCOVery, which includes modules through which patients receive indications of physical exercises, diet, and lifestyle, as well as training the amygdala and the insula through a program of neuroplasticity, mindfulness, and alternative nostril breathing [189,190]. In some cases, low-dose stimulants (e.g., methylphenidate or modafinil) can be considered if cognitive symptoms are severe and impairing [191]. These medications can help with attention and alertness, although they should be used cautiously and under medical supervision. Selective serotonin reuptake inhibitors (SSRIs) such as sertraline, fluoxetine, and escitalopram are commonly used for depression and anxiety. SSRIs are often the first-line treatment for these conditions in L-C19 patients [172,173,174,191]. Serotonin–norepinephrine reuptake inhibitors (SNRIs) like venlafaxine and duloxetine may also be effective for both depression and anxiety and can be considered when SSRIs are not effective [172,173]. In cases of treatment-resistant depression or patients experiencing significant sleep disturbances alongside mood symptoms, mirtazapine or tricyclic antidepressants can be used [128]. Benzodiazepines (e.g., lorazepam and diazepam) can be used in the short term for acute anxiety symptoms, but their use is limited due to the risk of dependence and tolerance [192]. They are typically avoided for long-term treatment. Buspirone is another anti-anxiety medication with a lower risk of dependence and can be considered in patients with generalized anxiety disorder [193]. SSRI antidepressants play an anti-inflammatory role by decreasing the production of cytokines, such as interleukins (IL) IL-1β, IL-6, IL-8, and IL-12 [175,194]. Fluoxetine, sertraline, paroxetine, and amitriptyline can interfere with the invasion of the central nervous system by the SARS-CoV-2 virus by inhibiting acid sphingomyelinase activity [195]. Inhibition of acid sphingomyelinase (FIASMA) blocks the conversion of sphingomyelin to ceramide, thus preventing SARS-CoV-2 from entering the host cell [196]. Antidepressants may also exert antiviral effects on SARS-CoV-2 through lysosomotropic properties [197]. Some antidepressants such as fluvoxamine reduce the serotonin load in platelets, thus inhibiting platelet activation and aggregation [198] and lowering the risk of thrombosis [199]. Melatonin could be effective in the treatment of long-term pressure from C-19 due to its antioxidant, anti-inflammatory, and antiapoptotic properties [178,179]. Mindfulness-based stress reduction (MBSR) and mindfulness-based cognitive therapy (MBCT) can help patients manage stress, anxiety, and depressive symptoms by increasing awareness and acceptance of their thoughts and emotions [176]. For patients with post-traumatic stress disorder (PTSD) related to their C-19 experience (e.g., hospitalization and ICU stay), trauma-focused therapies can help process trauma and reduce PTSD symptoms [169]. Eye movement desensitization and reprocessing (EMDR) can be used to help patients process traumatic memories and reduce PTSD symptoms [200]. Psychodynamic therapy and exposure therapy can also be beneficial for patients with PTSD [201]. Educating patients about good sleep habits is often the first step in managing sleep disturbances. This includes maintaining a regular sleep schedule, creating a comfortable sleep environment, and avoiding caffeine or heavy meals before bedtime [177,202]. Cognitive behavioral therapy for insomnia (CBT-I) is an evidence-based intervention that addresses the cognitive and behavioral factors that perpetuate insomnia [202]. It helps patients with sleep issues establish healthy sleep patterns without the need for medication. If non-pharmacological interventions are insufficient, medications such as melatonin, antihistamines, or low-dose tricyclic antidepressants (e.g., amitriptyline) can be prescribed for sleep disturbances [203]. Z-drugs like zolpidem or eszopiclone are sometimes used for short-term insomnia, although they carry the risk of dependence [173,183,204]. Vitamin D, B-group vitamins, and iron can be supplemented if deficiencies are identified. Some individuals with L-C19 have been shown to have low levels of these nutrients, which can contribute to fatigue [183,205]. Coenzyme Q_10_ (CoQ_10_) and N-acetylcysteine (NAC) have been explored for their potential to reduce fatigue and improve energy levels, although the evidence is still emerging [206,207]. There is ongoing research into the role of antiviral medications or immunomodulatory drugs to address the underlying pathophysiological mechanisms of L-C19, such as viral persistence or immune dysregulation. The use of nirmatrelvir/ritonavir during acute infection reduces the risk of prolonged C-19 [208]. Studies carried out in Japan have presented data about a new protease inhibitor, ensitrelvir, which is effective in the treatment of patients with chronic C-19 [209]. Although these treatments are not yet standard for neuropsychiatric symptoms, they may be considered in specific cases. If the development of an L-C19 is related to the persistence of the virus, the administration of an antiviral such as nirmatrelvir/ritonavir, remdesivir, or molnupiravir could block active replication, but at the same time, it might not have an effect on a stable and persistent reservoir [210,211]. Clinical research has shown that administering a nirmatrelvir/ritonavir drug combination upon the first symptoms of C-19 has the potential to decrease L-C19 development. Research conducted using a retrospective cohort design has shown that this antiviral medication decreases the risk of developing L-C19 symptoms in patients who face high medical risks [212]. Some research studies conducted on nirmatrelvir/ritonavir treatment have not established any substantial decrease in L-C19 symptom manifestation [213,214]. The analyzed retrospective cohort study found no meaningful distinction in the emergence of L-C19 symptoms between groups that received treatment versus those who did not receive it. Nevertheless, the nirmatrelvir/ritonavir treatment group demonstrated lower rates of brain fog and chest pain symptoms. The impact of remdesivir on L-C19 is not well-established. Research into the benefits of these drugs from the viewpoint of L-C19 prevention has shown conflicting results. Evidence from the systematic review failed to produce clear results about remdesivir’s success rate in preventing the emergence of L-C19 symptoms [214]. Applied reviewers call for newer investigations into this matter. Research on molnupiravir effectiveness against L-C19 has shown limited availability [215]. Preliminary findings suggest a modest reduction in L-C19 risk, but the data are not robust enough to draw definitive conclusions. Some patients may receive monoclonal antibody therapy (e.g., casirivimab–imdevimab) to help manage persistent symptoms related to immune response or ongoing viral replication [61,216]. The protease inhibitors lopinavir/ritonavir and darunavir/ritonavir, as well as favipiravir, a strong inhibitor of RNA-dependent RNA-polymerase, were initially used in the treatment of SARS-CoV-2 infection but are no longer included in current treatment protocols, either due to a lack of treatment efficiency or, as in the case of favipiravir, due to important adverse reactions [217]. Scientific research has shown that plant-based phytochemicals demonstrate promising efficiency as antiviral agents against C-19. Natural compounds from plants display antiviral effects by attacking different phases within the virus life cycle, starting from entry to replication and immune responses. The zinc finger of the main protease Mpro and viral entry processes are inhibited by antiviral flavonoids from fruits, vegetables, and tea, together with specific compounds like quercetin, kaempferol, and hesperidin. Scientific research has investigated the potential viral replication blockage in alkaloids through studies of lycorine and berberine. Two antiviral terpenoids include curcumin extracted from turmeric and glycyrrhizin derived from licorice because these compounds suppress inflammation and block viral entry into cells. Green tea contains epigallocatechin gallate (found in epigallocatechin gallate), while resveratrol exists in grapes, and both antiviral compounds show effects on viral proteins and stimulate immune response. Plant-derived compounds known as saponins improve immune system response and directly combat viruses. Scientists have conducted various in silico, in vitro, and in vivo research on these phytochemicals; yet, further clinical trials must prove their safety alongside their ability to treat L-C19 [218]. Some studies have investigated the effects of vortioxetine, glucosaminyl muramyl dipeptide (called licopid), leronlimab (a monoclonal antibody), and actovegin (derived from ultrafiltered calf blood) on the recovery of cognitive deficits in patients with L-C19, but these studies require confirmation by further research [167,218,219,220]. Some patients with L-C19 may have intestinal dysbiosis, which would contribute to the emergence of neuropsychiatric manifestations. The use of symbiotics to restore the diversity of the intestinal microbiome could help reduce fatigue and increase the ability to concentrate but not increase the quality of life [221,222]. Active high-definition transcranial direct current stimulation, photobiomodulation, and hyperbaric oxygen have been used in some studies for neuropsychiatric manifestations, but there is no high-certainty evidence that these methods would benefit patients with L-C19 [223,224,225]. Patients experiencing significant fatigue benefit from pacing (gradual increases in activity level without overexertion) and energy management strategies [181]. This includes balancing rest and activity, avoiding triggers of fatigue, and gradually increasing physical activity as tolerated [180]. Gradual exercise programs such as low-impact aerobic exercise (e.g., walking and swimming) can help alleviate fatigue and improve mood over time [170]. A tailored physical rehabilitation program may help with post-exertional malaise (PEM) and reconditioning after a period of inactivity [182]. L-C19 is associated with neuropathic pain, which can have central or peripheral etiologies, and has been attributed to peripheral neuropathy [185,226]. SARS-CoV-2 infection is also associated with demyelinating polyneuropathies, such as Guillain–Barré syndrome [227]. The treatment options for neuropathy related to C-19 involve a short course of steroids and intravenous immunoglobulin [228]. Gabapentinoids and antidepressants can also be tried, but many patients may improve without intervention [187].

The evidence from studies indicates that eicosapentaenoic acid (EPA) and docosahexaenoic acid (DHA), together with other long-chain omega-3 polyunsaturated fatty acids, could provide therapeutic effects for patients suffering from L-C19 symptoms [229]. Scientific evidence reveals that these benefits occur because omega-3 has anti-inflammatory functions and affects psychoneuroimmune system modulation [230]. The consumption of omega-3 polyunsaturated fatty acids leads to decreased systemic inflammation because the fats lower the production rates of pro-inflammatory cytokines. Research indicates that taking these supplements helps to reduce ongoing inflammatory symptoms that occur after C-19 infection [229,230]. Omega-3 has a regulatory effect on the HPA axis that both controls stress reactions and operates the immune system. The regulation of this axis by omega-3 fatty acids might help reduce the neuropsychiatric symptoms that occur during L-C19, especially depression and anxiety symptoms [231]. Proponents of omega-3 supplementation tested this therapy on unvaccinated C-19 patients with moderate illness in a randomized and double-blinded clinical trial [232]. New research demonstrates that omega-3 supplementation produced major positive effects on patients’ metabolic and inflammatory markers, thereby providing evidence for symptom control of C-19. Research on omega-3 supplementation for healthcare workers with L-C19 symptoms showed no substantial changes in their experienced symptoms based on statistical analysis [233]. The efficacy of omega-3 seems to differ from one person to another; therefore, scientists need to conduct additional studies to prove their definite medical benefits.

## 8. Impact on Quality of Life

The neuropsychiatric manifestations of L-C19 can have serious consequences on the quality of life of the people affected. Research studies have applied standard evaluation tools, including SF-36 and EQ-5D, to measure how L-C19 affects patients’ health-related quality of life (HRQoL). A prospective cross-sectional study examined quality of life (QoL) using SF-36 questionnaires to measure outcomes between patients with L-C19 and healthy participants. Study participants with L-C19 reported significant declines in their physical functioning, together with a reduced ability to perform physical roles and a diminished overall quality of life [234]. L-C19 patients show significantly decreased physical health measurements on the SF-36 relative to general population scores based on data from research studies [235]. A study evaluated the HRQoL of L-C19 patients by using EQ-5D-5L as its assessment instrument. The results showed that most patients reported limitations in daily activities, accompanied by pain, discomfort, and anxiety [236]. The EPICOVID-AP21 study investigated the prevalence of and factors associated with LC-19 symptoms among primary care patients. The study found that a significant proportion of individuals experienced persistent symptoms after acute COVID-19 infection. Common LC-19 symptoms identified included fatigue, cognitive disturbances, and respiratory issues. Factors such as age, gender, and the severity of the initial infection were associated with an increased likelihood of developing LC-19. The study emphasizes the importance of recognizing and addressing LC-19 in primary care settings to provide appropriate management and support for affected patients [237]. Another study investigated the impact of COVID-19 on patients’ functional status, physical activity levels, fatigue, and quality of life [238]. The researchers compared the level of physical activity and QoL in individuals with LC-19 compared to patients who did not develop persistent symptoms after the period of acute COVID-19. The findings revealed that 95% of patients in the LC-19 group had severe functional limitations compared to the group without symptoms after acute COVID-19. These results highlight the significant and prolonged impact of LC-19 on patients’ daily lives and well-being.

The impact on quality of life is multi-faceted, affecting various aspects of daily functioning and well-being. Cognitive impairments can significantly negatively impact work and academic performance and daily responsibilities, including managing finances, meeting personal responsibilities, and dealing with personal interactions, which can result in frustration, embarrassment, and decreased competence [239,240]. Anxiety and depression disrupt people’s daily lives, preventing them from fulfilling normal activities and connecting with others, thus isolating and disempowering them [241]. Sleep disturbances and fatigue can both result from these mood disturbances and reduce life satisfaction [137]. Fatigue can be one of the most disabling aspects of L-C19, rendering it impossible for a person to carry out basic daily activities, be social or engage in recreational pursuits, and even manage their own personal care [14]. When people become this tired, they may find themselves dependent on others, thus becoming less independent and experiencing lower self-worth [242]. Since poor sleep quality can exacerbate fatigue and cognitive issues, worsened health leads to a cycle of poor health. Sleep problems can also lead to mood and overall well-being problems and can affect physical and mental health alike [136,243]. This sensory bombardment is so overwhelming that it can cause irritability, leads children to avoid engaging in human environments, and interferes with their ability to function in “real-world” settings [244]. If this occurs, social isolation and an inability to work or live in community life may result. Flashbacks, nightmares, extra vigilance, and emotional numbing occur in people suffering from PTSD and can take a toll on their ability to perform daily activities and on their day-to-day mental health. It may also cause them to be afraid of reinfection or lead to trouble interacting with other people or leaving home [245]. Feeling lonely and depressed due to social isolation disconnects people from the emotional support offered by friends and family [246]. A lack of social interaction can also contribute to purposelessness of life and lower overall satisfaction with life [247]. Financial stability and career prospects can be affected in the long term, causing stress and feelings of anxiety, and can lead to overall feelings of non-accomplishment. Returning to pre-C-19 work routines or adjusting to new limitations may be difficult for people [248]. A person with these symptoms feels dragged down, not only by physical symptoms but also by neuropsychiatric ones, which pile up and worsen the overall burden on a person’s quality of life. People who are not able or are not required to engage much in physical activities or to seek frequent medical care are likely to be frustrated by reduced independence [249]. These issues can worsen stress and anxiety and bring more mental health problems. People with L-C19 often need support from family and caregivers who may themselves be stressed or burnt out. The need for caregiving can strain relationships, and the emotional burden on family members can negatively affect their own well-being. The caregiver’s quality of life may decrease due to fatigue, emotional strain, and financial concerns [250]. The EPICOVID-AP21 study investigated the prevalence and factors associated with LC-19 symptoms among primary care patients. The study found that a significant proportion of individuals experienced persistent symptoms after acute COVID-19 infection. Common LC-19 symptoms identified included fatigue, cognitive disturbances, and respiratory issues. Factors such as age, gender, and the severity of the initial infection were associated with an increased likelihood of developing LC-19. The study emphasizes the importance of recognizing and addressing LC-19 in primary care settings to provide appropriate management and support for affected patients [236]. Another study investigated the impact of COVID-19 on patients’ functional status, physical activity and fatigue levels, and quality of life [237]. The researchers compared the level of physical activity and QoL in individuals with LC-19 to those of patients who did not develop persistent symptoms after the period of acute COVID-19. The findings revealed that 95% of patients in the LC-19 group had severe functional limitations compared to the group without symptoms after acute COVID-19. These results highlight the significant and prolonged impact of LC-19 on patients’ daily lives and well-being.

## 9. Impact on the Quality of Life of Children

Together with their family, children living with L-C19 neuropsychiatric manifestations may see their quality of life impacted, and although this may be different in some ways to adults, the unique developmental and social needs of children must be taken into account [251]. L-C19 may cause cognitive, emotional, and behavioral symptoms in children, which in turn affect their physical, psychological, and social interactions [252]. These symptoms make it difficult for them to function during school, play, or peer socializing [253]. Memory problems, trouble concentrating, and problems processing information might be some of the cognitive difficulties children with L-C19 have. This is a phenomenon they commonly call brain fog, which is related to problems with attention, learning, and executive functioning [254]. Academic impairment due to cognitive disorders negatively affects students’ grades and overall participation. Struggling academically may be frustrating, involving feelings of being overwhelmed or embarrassed [255]. This can reduce achievement, increase stress levels, and result in feelings of inadequacy and failure. Children with L-C19 can develop some mood disorders, such as anxiety, depression, irritability, or mood swings [256,257]. Battling with physical symptoms such as fatigue or difficulty breathing makes them overly worried, sad, or fearful. For children, anxiety and depression can lead to withdrawal from social activities, losing interest in things that previously interested them, and feeling disconnected from peers [258,259]. In these mood disorders, emotional distress occurs, which is associated with problems with concentration in school or recreational activities and with family relationships [259]. Feelings of social withdrawal and isolation may increase loneliness. One of the most common symptoms of L-C19 in children is persistent fatigue. Fatigue can be disproportionate to any physical activity, and children can become worn out and unable to cope with normal routines [260]. The fatigue can be so severe that it interferes with a child going to school, participating in extracurricular activities, or even playing with friends. Extra rest may be needed, which interferes with their daily schedule. Fatigue may also lead to irritability and difficulty concentrating, which aggravates academic and social performance [261,262]. Sleep problems such as insomnia, not being able to stay asleep, or disrupted sleep cycles are common in children with L-C19. Fatigue and cognitive problems become worse because of sleep disturbances [263,264]. Poor sleep quality is associated with an impact on mood, attention, and behavior, which can impact a child’s capability to function at school and in a social setting [265]. It can also increase the chances of irritability, emotional dysregulation, and anxiety if a person does not get enough sleep. Children with L-C19 may demonstrate some increased sensitivity to light, sound, or touch and altered sensory perceptions [266]. Children who experience sensory overload can find ordinary environments uncomfortable or overwhelming and may, therefore, avoid social situations, classrooms, or public places. Sensory sensitivities can cause irritability, anxiety, and reluctance to participate in typical daily functioning, making it nearly impossible to go to school, play sports, or be involved in family activities [267]. Changes in behavior can present as neuropsychiatric symptoms due to the increased levels of irritability and aggression, mood swings, or emotional outbursts. These behaviors may be related to frustration from cognitive limits, fatigue, or emotional distress [268]. Behavioral changes can strain relationships with family members, peers, and teachers. Such children may have problems following routine expectations at home and in school, resulting in conflicts or social difficulties. These behavioral shifts also cause emotional isolation or feelings of being misunderstood. Children with L-C19 often have cognitive difficulties, mood changes, and physical symptoms that can lead to social withdrawal [269]. They may be separated from their classmates or be unable to participate in group activities [244]. Social isolation can have severe implications for a child’s sense of belonging and emotional development. They may have trouble making friends, joining school activities, or engaging in social activities. This can lead to loneliness, low self-esteem, and an increased risk of depression or anxiety [270]. Disruption can alienate children from those in their peer group, creating a feeling of “otherness” and a shrinking of the feeling of normalcy. If the child is not engaged in extracurricular activities, they may not be able to build social skills, hobbies, and a sense of accomplishment. Children with L-C19 might have cognitive, physical, or emotional challenges that prevent them from going to school or involving in extracurricular activities [271]. They may have difficulty concentrating and feel unrested, making it nearly impossible for them to attend classes or engage in sports and hobbies [272]. The academic progress and social development of a child could be affected by missing out on educational and social opportunities [271,273]. The neuropsychiatric symptoms of L-C19 not only impact the child but they can also be very emotionally burdensome on parents and caregivers who have to adapt to the changing needs of the affected child [272]. The child’s ongoing health problems may be stressful, frustrating, and worrisome for families. It can mean more caregiving duties for parents, financial stress, and emotional fatigue as a result of doing everything themselves. In some situations, caregivers taking on additional responsibility could alter family dynamics. As a result, families of children and adults may experience negative emotional effects. These neuropsychiatric symptoms of L-C19 typically coexist with other physical symptoms, like muscle pain, headaches, or gastrointestinal issues, which compound the neuropsychiatric symptoms [274,275]. Physical and neuropsychiatric symptoms can combine to make even ordinary things harder. Physical play or sports, which are important for both physical and social development, may be performed less among children. Mental and physical sequelae have a continuous impact on a child’s well-being.

## 10. Future Directions

As our understanding of L-C19 continues to evolve, early recognition, proper diagnosis, and tailored treatment approaches will be essential to help manage symptoms and improve the quality of life of affected individuals. Further research into the pathophysiology, diagnostic criteria, and treatment strategies for L-C19 neuropsychiatric manifestations is needed to provide effective interventions and support for this complex and often debilitating condition. Further research directions should involve the identification of biomarkers specific to L-C19, particularly as they relate to neuroinflammation or autonomic dysfunction, and an understanding of how C-19 affects brain structure and function using MRI and other neuroimaging techniques. Comparisons with other post-viral syndromes, including chronic fatigue syndrome, will help to find more effective treatments for L-C19. While there is some evidence to suggest that antiviral treatments during the acute phase of C-19 may influence the development of L-C19, the findings are mixed and not definitive. Further rigorous studies are needed to clarify the effectiveness of these antivirals in preventing or treating L-C19.

## 11. Conclusions

The neuropsychiatric manifestations of L-C19 represent a significant and growing health concern, with wide-ranging effects on the emotional and cognitive well-being of individuals. This review showed that combining screening tools, neuropsychological assessments, laboratory tests, and neuroimaging helps confirm the diagnosis and rule out other conditions. The data obtained from the literature revealed that, due to the complexity of L-C19, a multidisciplinary approach is often necessary to ensure that patients receive comprehensive care for their cognitive and mental health symptoms. We also found that L-C19 neuropsychiatric manifestations negatively impact several domains of an individual’s life, adversely affecting their quality of life. The reviewed studies demonstrated that people with L-C19 may experience cognitive, emotional, and social challenges that make it difficult to perform day-to-day functions, participate in meaningful activities, or maintain a sense of control or accomplishment. Through measurements obtained from validated tools, including SF-36 and EQ-5D, the research findings confirmed that L-C19 significantly affects HRQoL. That is not to say that these effects affect only the individuals experiencing them; families, caregivers, and communities are also affected. Therefore, L-C19 requires comprehensive support, including mental health interventions, physical rehabilitation, and societal awareness of the long-term effects of the disease.

## Figures and Tables

**Table 1:** Main neurological manifestations reported by adults with L-C19.

Manifestations	Population (n =)	Observations	References
Depression, post-traumatic symptoms, and anxiety	282,711	Interventions should be focused on reducing the inflammatory process (reconditioning or mindfulness).	[114]
Ischemic and hemorrhagic stroke, cognition and memory disorders, and peripheral nervous system disorders	154,068	The risks were evident in people who were not hospitalized during the acute phase of the disease.	[115]
Chronic fatigue, pain, sleep disorders, and concentration problems	25,268	Fatigue was reported by women more than men.	[116]
Fatigue, post-exertional malaise, and cognitive dysfunction	3762	Seven months after the C-19 acute phase, many patients had not returned to previous levels of work and continued to experience significant symptom burden.	[16]
Anxiety, depression, and sleep disorders	606	Patients with neurological complications during index hospitalization had significantly worse 6-month functional outcomes than those without them.	[19]
Brain fog, headache, and dizziness	600	Anosmia, dysgeusia, and myalgia were more frequent in the non-hospitalized patients than in patients who were hospitalized in the C-19 acute phase (59 vs. 39).	[16]
Headache, fatigue, muscle aches/myalgia, articular pains, cognitive impairment, loss of concentration, and loss of smell	507	Subjects with L-C19 presented a lower self-esteem and a lower level of quality of life.	[117]
Fatigue, myalgia, sleep disorders, cognitive impairment, hyposmia, and dysgeusia	303	A higher prevalence of persisting symptoms was noted in older age groups (47–58 years) as well as in female participants.	[118]
Fatigue, memoryand sleep disorders	165	Age, the presence of comorbidities, and the severity of acute C-19 were independent factors.	[119]
Fatigue, headache, sleep disorders, and sensitivity alterations	103	Most of the symptoms started in the acute phase of C-19.	[120]
Fatigue, depression, and autoimmune disease	100	Non-hospitalized L-C19 patients experienced persistent fatigue and brain fog, which affected their quality of life.	[121]

**Table 2:** Diagnostic approach for neuropsychiatric manifestations of L-C19.

Diagnostic Method	Purpose	Examples of Tools/Tests
Clinical Interview	Assess symptom onset, duration, and severity.	Structured interviews and medical history review
Self-Reported Questionnaires	Screen for cognitive and psychiatric symptoms.	MoCA, MMSE, CFQ, HADS, PHQ-9, GAD-7, BDI, and PCL-5
Neuropsychological Testing	Evaluate cognitive functions (memory, attention, etc.).	Paper-and-pencil tasks and computerized assessments
Laboratory Tests	Rule out alternative causes (e.g., vitamin deficiencies).	TSH, T4, Vitamin B12, Vitamin D, CBC, and Liver/Kidney function
Brain Imaging	Detect structural or functional abnormalities.	MRI and CT scans
Electroencephalogram (EEG)	Assess brain activity in cases of seizures or abnormalities.	EEG recording of electrical activity
Autoimmune and Other Biomarkers	Exclude autoimmune disorders mimicking symptoms.	Rheumatoid factor and anti-nuclear antibodies
Multidisciplinary Assessment	Comprehensive evaluation by specialists.	Neurologists, psychiatrists, and sleep specialists

**Table 3:** Standardized questionnaires useful in the management of L-C19 cognitive impairments.

Type of Test	Usefulness	References
Montreal Cognitive Assessment (MoCA)	Screens for cognitive impairment, especially mild dysfunction.	[145,146,147,148,149,150,151]
Mini-Mental State Examination (MMSE)	Assesses cognitive function, focusing on memory, attention, and orientation.	[145,146,147,148,149,150,151]
Cognitive Failures Questionnaire (CFQ)	Evaluates everyday cognitive failures like forgetfulness and attention problems.	[145,146,147,148,149,150,151]
Hospital Anxiety and Depression Scale (HADS)	Screens for anxiety and depression symptoms in clinical settings.	[145,146,147,148,149,150,151]
Patient Health Questionnaire (PHQ-9)	Assesses the severity of depressive symptoms.	[146,149,151,152,153,154,155]
Generalized Anxiety Disorder-7 (GAD-7)	Screens for generalized anxiety disorder symptoms.	[146,149,151,152,153,154,155]
Beck Depression Inventory (BDI)	Measures the severity of depressive symptoms through self-reporting.	[146,149,151,152,153,154,155]
PTSD Checklist for DSM-5 (PCL-5)	Screens for and helps diagnose PTSD symptoms, especially after trauma or severe illness.	[156]
Pittsburgh Sleep Quality Index (PSQI)	Assesses sleep quality and disturbances common in L-C19 patients.	[156]

**Table 4:** Neuropsychiatric manifestations of L-C19 and treatment approaches.

Symptom Category	Main Manifestations	Conventional Treatment	Alternative Therapies	References
Cognitive issues	Brain fog and memory problems	Cognitive rehabilitation, stimulants, actovegin, exercise, and mindfulness.	Nootropic supplements (omega-3 and ginkgo biloba), meditation, and acupuncture.	[166,167,168,169,170]
Mood disorders	Anxiety, depression, and PTSD-like symptoms	SSRIs/SNRIs, psychotherapy (CBT), and lifestyle modifications.	Adaptogenic herbs (ashwagandha and rhodiola), mindfulness-based stress reduction (MBSR), and breathwork techniques.	[166,169,170,171,172,173,174,175,176]
Fatigue and sleep disturbances	Fatigue and insomnia	Sleep hygiene, cognitive behavioral therapy for insomnia (CBT-I), melatonin, graded exercise therapy, and cognitive pacing.	Yoga, Tai Chi, acupuncture, and herbal supplements (valerian root, magnesium, and ashwagandha).	[169,177,178,179,180,181,182]
Neuropathic	Headaches, paresthesia, dizziness, and autonomic dysfunctions	Neuromodulators (gabapentin and amitriptyline), hydration, and autonomic rehabilitation.	Acupuncture, biofeedback, vagus nerve stimulation, and anti-inflammatory diets.	[183,184,185,186]

## Data Availability

The original contributions presented in this study are included in the article. Further inquiries can be directed to the corresponding author.

## Abbreviations

The following abbreviations are used in this manuscript:

ACE2	Angiotensin-converting enzyme 2
ANS	Autonomic nervous system
ARDS	Acute respiratory distress syndrome
BBB	Blood–brain barrier
BDI	Beck Depression Inventory
C-19	COVID-19
CBC	Complete blood count
CBT	Cognitive behavioral therapy
CBT-I	Cognitive behavioral therapy for insomnia
CFS	Chronic fatigue syndrome
CFQ	Cognitive Failures Questionnaire
CIRCI	Critical illness-related corticosteroid insufficiency
CNS	Central nervous system
CoQ_10_	Coenzyme Q_10_
DHA	Docosahexaenoic acid
EAS	Extended autonomic system
EEG	Electroencephalogram
EMDR	Eye movement desensitization and reprocessing
EPA	Eicosapentaenoic acid
FIASMA	Inhibition of acid sphingomyelinase
GAD	Generalized anxiety disorder
GAD-7	Generalized Anxiety Disorder-7
HADS	Hospital Anxiety and Depression Scale
HPA	Hypothalamic–pituitary–adrenal
HRQoL	Health-related quality of life
QoL	quality of life
ICU	Intensive care unit
IL	Interleukins
L-C19	Long COVID-19
MBCT	Mindfulness-based cognitive therapy
MBSR	Mindfulness-based stress reduction
MCI	Mild cognitive impairment
MDD	Major depressive disorder
MMSE	Mini-Mental State Examination
MoCA	Montreal Cognitive Assessment
MRI	Magnetic resonance imaging
NAC	N-acetylcysteine
OCD	Obsessive–compulsive disorder
PASC	Post-acute sequelae of SARS-CoV-2 infection
PCL-5	PTSD Checklist for DSM-5
PEM	Post-exertional malaise
PHQ-9	Patient Health Questionnaire
POTS	Postural orthostatic tachycardia syndrome
PTSD	Post-traumatic stress disorder
PSQI	Pittsburgh Sleep Quality Index
SSRI	Selective serotonin reuptake inhibitor
TSH	Thyroid-Stimulating Hormone
T4	Thyroxine

## References

[1] Kabir M.T., Uddin M.S., Hossain M.F., Abdulhakim J.A., Alam M.A., Ashraf G.M., Bungau S.G., Bin-Jumah M.N., Abdel-Daim M.M., Aleya L. (2020). nCOVID-19 Pandemic: From Molecular Pathogenesis to Potential Investigational Therapeutics. Front. Cell Dev. Biol..

[2] Cocuz M.-E., Cocuz I.G., Rodina L., Tataranu E., Caliman-Sturdza O.A., Filip F. (2024). Treatment with Remdesivir of Children with SARS-CoV-2 Infection: Experience from a Clinical Hospital in Romania. Life.

[3] World Health Organization Home Page. A Clinical Case Definition of Post COVID-19 Condition by a Delphi Consensus, 6 October 2021. https://www.who.int/publications/i/item/WHO-2019-nCoV-Post_COVID-19_condition-Clinical_case_definition-2021.1.

[4] Soriano J.B., Murthy S., Marshall J.C., Relan P., Diaz J.V. (2021). A clinical case definition of post-COVID-19 condition by a Delphi consensus. Lancet Infect. Dis..

[5] Ely E.W., Brown L.M., Fineberg H.V. (2024). Long Covid Defined. N. Engl. J. Med..

[6] Nalbandian A., Sehgal K., Gupta A., Madhavan M.V., McGroder C., Stevens J.S., Cook J.R., Nordvig A.S., Shalev D., Sehrawat T.S. (2021). Post-acute COVID-19 syndrome. Nat. Med..

[7] Lopez-Leon S., Wegman-Ostrosky T., Perelman C., Sepulveda R., Rebolledo P.A., Cuapio A., Villapol S. (2021). More than 50 long-term effects of COVID-19: A systematic review and meta-analysis. Sci. Rep..

[8] Seeßle J., Waterboer T., Hippchen T., Simon J., Kirchner M., Lim A., Müller B., Merle U. (2022). Persistent Symptoms in Adult Patients 1 Year After Coronavirus Disease 2019 (COVID-19): A Prospective Cohort Study. Clin. Infect. Dis..

[9] Chen X., Laurent S., Onur O.A., Kleineberg N.N., Fink G.R., Schweitzer F., Warnke C. (2021). A systematic review of neurological symptoms and complications of COVID-19. J. Neurol..

[10] Wang H.-Y., Li X.-L., Yan Z.-R., Sun X.-P., Han J., Zhang B.-W. (2020). Potential neurological symptoms of COVID-19. Ther. Adv. Neurol. Disord..

[11] Carod-Artal F., García-Moncó J. (2021). Epidemiology, pathophysiology, and classification of the neurological symptoms of post-COVID-19 syndrome. Neurol. Perspect..

[12] Bungenberg J., Humkamp K., Hohenfeld C., Rust M.I., Ermis U., Dreher M., Hartmann N.K., Marx G., Binkofski F., Finke C. (2022). Long COVID-19: Objectifying most self-reported neurological symptoms. Ann. Clin. Transl. Neurol..

[13] Navis A. (2023). A Review of Neurological Symptoms in Long COVID and Clinical Management. Semin. Neurol..

[14] Aiyegbusi O.L., Hughes S.E., Turner G., Rivera S.C., McMullan C., Chandan J.S., Haroon S., Price G., Davies E.H., Nirantharakumar K. (2021). Symptoms, complications and management of long COVID: A review. J. R. Soc. Med..

[15] Orsucci D., Ienco E.C., Nocita G., Napolitano A., Vista M. (2020). Neurological features of COVID-19 and their treatment: A review. Drugs Context.

[16] Davis H.E., Assaf G.S., McCorkell L., Wei H., Low R.J., Re’Em Y., Redfield S., Austin J.P., Akrami A. (2021). Characterizing long COVID in an international cohort: 7 months of symptoms and their impact. eClinicalMedicine.

[17] Boesl F., Audebert H., Endres M., Prüss H., Franke C. (2021). A Neurological Outpatient Clinic for Patients with Post-COVID-19 Syndrome—A Report on the Clinical Presentations of the First 100 Patients. Front. Neurol..

[18] Mao L., Jin H., Wang M., Hu Y., Chen S., He Q., Chang J., Hong C., Zhou Y., Wang D. (2020). Neurologic Manifestations of Hospitalized Patients with Coronavirus Disease 2019 in Wuhan, China. JAMA Neurol..

[19] Frontera J.A., Yang D., Lewis A., Patel P., Medicherla C., Arena V., Fang T., Andino A., Snyder T., Madhavan M. (2021). A prospective study of long-term outcomes among hospitalized COVID-19 patients with and without neurological complications. J. Neurol. Sci..

[20] Taquet M., Sillett R., Zhu L., Mendel J., Camplisson I., Dercon Q., Harrison P.J. (2022). Neurological and psychiatric risk trajectories after SARS-CoV-2 infection: An analysis of 2-year retrospective cohort studies including 1,284,437 patients. Lancet Psychiatry.

[21] Pandharipande P., Roberson S.W., Harrison F.E., Wilson J.E., Bastarache J.A., Ely E.W. (2023). Mitigating neurological, cognitive, and psychiatric sequelae of COVID-19-related critical illness. Lancet Respir. Med..

[22] Greene T., El-Leithy S., Billings J., Albert I., Birch J., Campbell M., Ehntholt K., Fortune L., Gilbert N., Grey N. (2022). Anticipating PTSD in severe COVID survivors: The case for screen-and-treat. Eur. J. Psychotraumatology.

[23] Chaudhary A.M.D., Musavi N.B., Saboor S., Javed S., Khan S., Naveed S. (2022). Psychosis during the COVID-19 pandemic: A systematic review of case reports and case series. J. Psychiatr. Res..

[24] Poole-Wright K., Guennouni I., Sterry O., Evans R.A., Gaughran F., Chalder T. (2023). Fatigue outcomes following COVID-19: A systematic review and meta-analysis. BMJ Open.

[25] Ceban F., Ling S., Lui L.M., Lee Y., Gill H., Teopiz K.M., Rodrigues N.B., Subramaniapillai M., Di Vincenzo J.D., Cao B. (2022). Fatigue and cognitive impairment in Post-COVID-19 Syndrome: A systematic review and meta-analysis. Brain Behav. Immun..

[26] Umesh A., Pranay K., Pandey R.C., Gupta M.K. (2022). Evidence mapping and review of long-COVID and its underlying pathophysiological mechanism. Infection.

[27] Peluso M.J., Deeks S.G. (2022). Early clues regarding the pathogenesis of long-COVID. Trends Immunol..

[28] Mohandas S., Jagannathan P., Henrich T.J., A Sherif Z., Bime C., Quinlan E., A Portman M., Gennaro M., Rehman J., Force R.M.P.T. (2023). Immune mechanisms underlying COVID-19 pathology and post-acute sequelae of SARS-CoV-2 infection (PASC). eLife.

[29] Klein J., Wood J., Jaycox J., Lu P., Dhodapkar R.M., Gehlhausen J.R., Tabachnikova A., Tabacof L., Malik A.A., Kamath K. (2022). Distinguishing features of Long COVID identified through immune profiling. medRxiv.

[30] Robinson-Agramonte M.A., Gonçalves C.-A., Noris-García E., Rivero N.P., Brigida A.L., Schultz S., Siniscalco D., García R.J.G. (2021). Impact of SARS-CoV-2 on neuropsychiatric disorders. World J. Psychiatry.

[31] Taquet M., Skorniewska Z., Hampshire A., Chalmers J.D., Ho L.-P., Horsley A., Marks M., Poinasamy K., Raman B., Leavy O.C. (2023). Acute blood biomarker profiles predict cognitive deficits 6 and 12 months after COVID-19 hospitalization. Nat. Med..

[32] Peluso M.J., Lu S., Tang A.F., Durstenfeld M.S., Ho H.-E., A Goldberg S., A Forman C., E Munter S., Hoh R., Tai V. (2021). Markers of Immune Activation and Inflammation in Individuals with Postacute Sequelae of Severe Acute Respiratory Syndrome Coronavirus 2 Infection. J. Infect. Dis..

[33] Merad M., Blish C.A., Sallusto F., Iwasaki A. (2022). The immunology and immunopathology of COVID-19. Science.

[34] Lavi E., Cong L. (2020). Type I astrocytes and microglia induce a cytokine response in an encephalitic murine coronavirus infection. Exp. Mol. Pathol..

[35] Mohamed M.S., Johansson A., Jonsson J., Schiöth H.B. (2022). Dissecting the Molecular Mechanisms Surrounding Post-COVID-19 Syndrome and Neurological Features. Int. J. Mol. Sci..

[36] Tremblay M.-E., Madore C., Bordeleau M., Tian L., Verkhratsky A. (2020). Neuropathobiology of COVID-19: The Role for Glia. Front. Cell. Neurosci..

[37] De Melo G.D., Lazarini F., Levallois S., Hautefort C., Michel V., Larrous F., Verillaud B., Aparicio C., Wagner S., Gheusi G. (2021). COVID-19–related anosmia is associated with viral persistence and inflammation in human olfactory epithelium and brain infection in hamsters. Sci. Transl. Med..

[38] Vonck K., Garrez I., De Herdt V., Hemelsoet D., Laureys G., Raedt R., Boon P. (2020). Neurological manifestations and neuro-invasive mechanisms of the severe acute respiratory syndrome coronavirus type 2. Eur. J. Neurol..

[39] Thye A.Y.-K., Law J.W.-F., Tan L.T.-H., Pusparajah P., Ser H.-L., Thurairajasingam S., Letchumanan V., Lee L.-H. (2022). Psychological Symptoms in COVID-19 Patients: Insights into Pathophysiology and Risk Factors of Long COVID-19. Biology.

[40] Desai I., Manchanda R., Kumar N., Tiwari A., Kumar M. (2021). Neurological manifestations of coronavirus disease 2019: Exploring past to understand present. Neurol. Sci..

[41] Jin Y., Yang H., Ji W., Wu W., Chen S., Zhang W., Duan G. (2020). Virology, Epidemiology, Pathogenesis, and Control of COVID-19. Viruses.

[42] Zubair A.S., McAlpine L.S., Gardin T., Farhadian S., Kuruvilla D.E., Spudich S. (2020). Neuropathogenesis and Neurologic Manifestations of the Coronaviruses in the Age of Coronavirus Disease 2019: A Review. JAMA Neurol..

[43] Choutka J., Jansari V., Hornig M., Iwasaki A. (2022). Unexplained post-acute infection syndromes. Nat. Med..

[44] Song E., Bartley C.M., Chow R.D., Ngo T.T., Jiang R., Zamecnik C.R., Dandekar R., Loudermilk R.P., Dai Y., Liu F. (2021). Divergent and self-reactive immune responses in the CNS of COVID-19 patients with neurological symptoms. Cell Rep. Med..

[45] Baig A.M. (2020). Deleterious Outcomes in Long-Hauler COVID-19: The Effects of SARS-CoV-2 on the CNS in Chronic COVID Syndrome. ACS Chem. Neurosci..

[46] Perrin P., Collongues N., Baloglu S., Bedo D., Bassand X., Lavaux T., Gautier-Vargas G., Keller N., Kremer S., Fafi-Kremer S. (2021). Cytokine release syndrome-associated encephalopathy in patients with COVID-19. Eur. J. Neurol..

[47] Lee M.-H., Perl D.P., Nair G., Li W., Maric D., Murray H., Dodd S.J., Koretsky A.P., Watts J.A., Cheung V. (2021). Microvascular Injury in the Brains of Patients with Covid-19. N. Engl. J. Med..

[48] Pretorius E., Venter C., Laubscher G.J., Kotze M.J., Oladejo S.O., Watson L.R., Rajaratnam K., Watson B.W., Kell D.B. (2022). Prevalence of symptoms, comorbidities, fibrin amyloid microclots and platelet pathology in individuals with Long COVID/Post-Acute Sequelae of COVID-19 (PASC). Cardiovasc. Diabetol..

[49] Gąsecka A., Borovac J.A., Guerreiro R.A., Giustozzi M., Parker W., Caldeira D., Chiva-Blanch G. (2021). Thrombotic Complications in Patients with COVID-19: Pathophysiological Mechanisms, Diagnosis, and Treatment. Cardiovasc. Drugs Ther..

[50] McAlpine L.S., Zubair A.S., Maran I., Chojecka P., Lleva P., Jasne A.S., Navaratnam D., Matouk C., Schindler J., Sheth K.N. (2021). Ischemic Stroke, Inflammation, and Endotheliopathy in COVID-19 Patients. Stroke.

[51] Zhou T., Sawano M., Arun A.S., Caraballo C., Michelsen T., McAlpine L.S., Bhattacharjee B., Lu Y., Khera R., Huang C. (2024). Internal Tremors and Vibrations in Long COVID: A Cross-Sectional Study. Am. J. Med..

[52] Abdennour L., Zeghal C., Dème M., Puybasset L. (2012). Interaction cerveau-poumon. Ann. Françaises D’anesthésie Et De Réanimation.

[53] Wong A.C., Devason A.S., Umana I.C., Cox T.O., Dohnalová L., Litichevskiy L., Perla J., Lundgren P., Etwebi Z., Izzo L.T. (2023). Serotonin reduction in post-acute sequelae of viral infection. Cell.

[54] Efstathiou V., Stefanou M.-I., Demetriou M., Siafakas N., Makris M., Tsivgoulis G., Zoumpourlis V., Kympouropoulos S.P., Tsoporis J.N., Spandidos D.A. (2022). Long COVID and neuropsychiatric manifestations (Review). Exp. Ther. Med..

[55] Attademo L., Bernardini F. (2020). Are dopamine and serotonin involved in COVID-19 pathophysiology?. Eur. J. Psychiatry.

[56] Smith S.M., Vale W.W. (2006). The role of the hypothalamic-pituitary-adrenal axis in neuroendocrine responses to stress. Dialog. Clin. Neurosci..

[57] Su Y., Yuan D., Chen D.G., Ng R.H., Wang K., Choi J., Li S., Hong S., Zhang R., Xie J. (2022). Multiple early factors anticipate post-acute COVID-19 sequelae. Cell.

[58] Jensterle M., Herman R., Janež A., Al Mahmeed W., Al-Rasadi K., Al-Alawi K., Banach M., Banerjee Y., Ceriello A., Cesur M. (2022). The Relationship between COVID-19 and Hypothalamic–Pituitary–Adrenal Axis: A Large Spectrum from Glucocorticoid Insufficiency to Excess—The CAPISCO International Expert Panel. Int. J. Mol. Sci..

[59] Alzahrani A.S., Mukhtar N., Aljomaiah A., Aljamei H., Bakhsh A., Alsudani N., Elsayed T., Alrashidi N., Fadel R., Alqahtani E. (2021). The Impact of COVID-19 Viral Infection on the Hypothalamic-Pituitary-Adrenal Axis. Endocr. Pract..

[60] Sahoo S.K., Sahoo S.K., Menon J.C., Menon J.C., Tripathy N., Tripathy N., Nayak M., Nayak M., Yadav S., Yadav S. (2024). Reversible central adrenal insufficiency in survivors of COVID-19: Results from a 24-month longitudinal study. Endocr. Connect..

[61] Iwasaki A., Putrino D. (2023). Why we need a deeper understanding of the pathophysiology of long COVID. Lancet Infect. Dis..

[62] Singh R., Rathore S.S., Khan H., Karale S., Chawla Y., Iqbal K., Bhurwal A., Tekin A., Jain N., Mehra I. (2022). Association of Obesity With COVID-19 Severity and Mortality: An Updated Systemic Review, Meta-Analysis, and Meta-Regression. Front. Endocrinol..

[63] Park J.-H., Park S., Kim N.-H., Lee Y., Chang Y., Song T.-J. (2024). Postural Orthostatic Tachycardia Syndrome Associated with COVID-19: A Narrative Review. Medicina.

[64] Guntur V.P., Nemkov T., de Boer E., Mohning M.P., Baraghoshi D., Cendali F.I., San-Millán I., Petrache I., D’alessandro A. (2022). Signatures of Mitochondrial Dysfunction and Impaired Fatty Acid Metabolism in Plasma of Patients with Post-Acute Sequelae of COVID-19 (PASC). Metabolites.

[65] Cheng W.-J., Shih H.-M., Su K.-P., Hsueh P.-R. (2023). Risk factors for poor COVID-19 outcomes in patients with psychiatric disorders. Brain Behav. Immun..

[66] Huang L., Yao Q., Gu X., Wang Q., Ren L., Wang Y., Hu P., Guo L., Liu M., Xu J. (2021). 1-year outcomes in hospital survivors with COVID-19: A longitudinal cohort study. Lancet.

[67] Lagadinou M., Kostopoulou E., Karatza A., Marangos M., Gkentzi D. (2021). The prolonged effects of COVID-A new “threat”?. Eur. Rev. Med. Pharmacol. Sci..

[68] Crook H., Raza S., Nowell J., Young M., Edison P. (2021). Long Covid—Mechanisms, Risk Factors, and Management. BMJ.

[69] Thakur K.T., Miller E.H., Glendinning M.D., Al-Dalahmah O., Banu M.A., Boehme A.K., Boubour A.L., Bruce S.S., Chong A.M., Claassen J. (2021). COVID-19 neuropathology at Columbia University Irving Medical Center/New York Presbyterian Hospital. Brain.

[70] Almeria M., Cejudo J.C., Sotoca J., Deus J., Krupinski J. (2020). Cognitive profile following COVID-19 infection: Clinical predictors leading to neuropsychological impairment. Brain Behav. Immun. Health.

[71] Zakia H., Pradana K., Iskandar S. (2023). Risk factors for psychiatric symptoms in patients with long COVID: A systematic review. PLoS ONE.

[72] Sykes D.L., Holdsworth L., Jawad N., Gunasekera P., Morice A.H., Crooks M.G. (2021). Post-COVID-19 Symptom Burden: What is Long-COVID and How Should We Manage It?. Lung.

[73] Gouraud C., Bottemanne H., Lahlou-Laforêt K., Blanchard A., Günther S., El Batti S., Auclin E., Limosin F., Hulot J.-S., Lebeaux D. (2021). Association Between Psychological Distress, Cognitive Complaints, and Neuropsychological Status After a Severe COVID-19 Episode: A Cross-Sectional Study. Front. Psychiatry.

[74] Taquet M., Dercon Q., Luciano S., Geddes J.R., Husain M., Harrison P.J. (2021). Incidence, co-occurrence, and evolution of long-COVID features: A 6-month retrospective cohort study of 273,618 survivors of COVID-19. PLoS Med..

[75] Seens H., Modarresi S., Fraser J., MacDermid J.C., Walton D.M., Grewal R. (2021). The role of sex and gender in the changing levels of anxiety and depression during the COVID-19 pandemic: A cross-sectional study. Women’s Health.

[76] Müller L., Di Benedetto S. (2023). Aged brain and neuroimmune responses to COVID-19: Post-acute sequelae and modulatory effects of behavioral and nutritional interventions. Immun. Ageing.

[77] Matias-Guiu J.A., Herrera E., González-Nosti M., Krishnan K., Delgado-Alonso C., Díez-Cirarda M., Yus M., Martínez-Petit Á., Pagán J., Matías-Guiu J. (2023). Development of criteria for cognitive dysfunction in post-COVID syndrome: The IC-CoDi-COVID approach. Psychiatry Res..

[78] Callender L.A., Curran M., Bates S.M., Mairesse M., Weigandt J., Betts C.J. (2020). The Impact of Pre-existing Comorbidities and Therapeutic Interventions on COVID-19. Front. Immunol..

[79] Roberts J., Pritchard A.L., Treweeke A.T., Rossi A.G., Brace N., Cahill P., MacRury S.M., Wei J., Megson I.L. (2021). Why Is COVID-19 More Severe in Patients With Diabetes? The Role of Angiotensin-Converting Enzyme 2, Endothelial Dysfunction and the Immunoinflammatory System. Front. Cardiovasc. Med..

[80] Fung M., Babik J.M. (2021). COVID-19 in Immunocompromised Hosts: What We Know So Far. Clin. Infect. Dis..

[81] Bigdelou B., Sepand M.R., Najafikhoshnoo S., Negrete J.A.T., Sharaf M., Ho J.Q., Sullivan I., Chauhan P., Etter M., Shekarian T. (2022). COVID-19 and Preexisting Comorbidities: Risks, Synergies, and Clinical Outcomes. Front. Immunol..

[82] Sakibuzzaman, Hassan A., Hayee S., Haque F.A., Bushra S.S., Maliha M., Tania M.K., Sadat A., Akter F., Mazumder T. (2022). Exacerbation of Pre-existing Neurological Symptoms With COVID-19 in Patients With Chronic Neurological Diseases: An Updated Systematic Review. Cureus.

[83] Brown E.E., Kumar S., Rajji T.K., Pollock B.G., Mulsant B.H. (2020). Anticipating and Mitigating the Impact of the COVID-19 Pandemic on Alzheimer’s Disease and Related Dementias. Am. J. Geriatr. Psychiatry.

[84] Astin R., Banerjee A., Baker M.R., Dani M., Ford E., Hull J.H., Lim P.B., McNarry M., Morten K., O’Sullivan O. (2023). Long COVID: Mechanisms, risk factors and recovery. Exp. Physiol..

[85] Phetsouphanh C., Darley D.R., Wilson D.B., Howe A., Munier C.M.L., Patel S.K., Juno J.A., Burrell L.M., Kent S.J., Dore G.J. (2022). Immunological dysfunction persists for 8 months following initial mild-to-moderate SARS-CoV-2 infection. Nat. Immunol..

[86] Ryan F.J., Hope C.M., Masavuli M.G., Lynn M.A., Mekonnen Z.A., Yeow A.E.L., Garcia-Valtanen P., Al-Delfi Z., Gummow J., Ferguson C. (2022). Long-term perturbation of the peripheral immune system months after SARS-CoV-2 infection. BMC Med..

[87] Jeffers A., Meehan A.A., Barker J., Asher A., Montgomery M.P., Bautista G., Ray C.M., Laws R.L., Fields V.L., Radhakrishnan L. (2022). Impact of Social Isolation during the COVID-19 Pandemic on Mental Health, Substance Use, and Home-lessness: Qualitative Interviews with Behavioral Health Providers. Int. J. Environ. Res. Public Health.

[88] Bertollo A.G., Braga G.d.C., Tonin P.T., Luzardo A.R., Bagatini M.D., Ignácio Z.M. (2023). The Impact of Stress from Social Isolation during the COVID-19 Pandemic on Psychiatric Disorders: An Analysis from the Scientific Literature. Brain Sci..

[89] Strausz S., Kiiskinen T., Broberg M., Ruotsalainen S., Koskela J., Bachour A., Gen F., Palotie A., Palotie T., Ripatti S. (2021). Sleep apnoea is a risk factor for severe COVID-19. BMJ Open Respir. Res..

[90] Meng S., Lu L., Yuan K., Yang D., Zhang I. (2022). Facing Sleep and Mental Health Problems in the COVID-19 Era: What Shall We Do?. Heart Mind.

[91] Pairo-Castineira E., Clohisey S., Klaric L., Bretherick A.D., Rawlik K., Pasko D., Walker S., Parkinson N., Fourman M.H., Russell C.D. (2020). Genetic mechanisms of critical illness in COVID-19. Nature.

[92] Taylor K., Pearson M., Das S., Sardell J., Chocian K., Gardner S. (2023). Genetic risk factors for severe and fatigue dominant long COVID and commonalities with ME/CFS identified by combinatorial analysis. J. Transl. Med..

[93] Lo Presti M., Beck D.B., Duggal P., Cummings D.A., Solomon B.D. (2020). The Role of Host Genetic Factors in Coronavirus Susceptibility: Review of Animal and Systematic Review of Human Literature. Am. J. Hum. Genet..

[94] Amo C., Almansour N., Harvey I.S. (2022). Physical Activity and Mental Health Declined during the Time of the COVID-19 Pandemic: A Narrative Literature Review. Int. J. Environ. Res. Public Health.

[95] Wanjau M.N., Möller H., Haigh F., Milat A., Hayek R., Lucas P., Veerman J.L. (2023). Physical Activity and Depression and Anxiety Disorders: A Systematic Review of Reviews and Assessment of Causality. AJPM Focus.

[96] Hansel T.C., Saltzman L.Y., Melton P.A., Clark T.L., Bordnick P.S. (2022). COVID-19 behavioral health and quality of life. Sci. Rep..

[97] Peluso M.J., Deveau T.-M., Munter S.E., Ryder D., Buck A., Beck-Engeser G., Chan F., Lu S., Goldberg S.A., Hoh R. (2023). Chronic viral coinfections differentially affect the likelihood of developing long COVID. J. Clin. Investig..

[98] Graham E.L., Clark J.R., Orban Z.S., Lim P.H., Szymanski A.L., Taylor C., DiBiase R.M., Jia D.T., Balabanov R., Ho S.U. (2021). Persistent neurologic symptoms and cognitive dysfunction in non-hospitalized Covid-19 “long haulers”. Ann. Clin. Transl. Neurol..

[99] Jennings G., Monaghan A., Xue F., Duggan E., Romero-Ortuño R. (2022). Comprehensive Clinical Characterisation of Brain Fog in Adults Reporting Long COVID Symptoms. J. Clin. Med..

[100] Talhari C., Criado P.R., Castro C.C.S., Ianhez M., Ramos P.M., Miot H.A. (2023). Prevalence of and risk factors for post-COVID: Results from a survey of 6958 patients from Brazil. Acad. Bras. Cienc..

[101] Van der Maaden T., Mutubuki E.N., de Bruijn S., Leung K.Y., Knoop H., Slootweg J., Tulen A.D., Wong A., van Hoek A.J., Franz E. (2023). Prevalence and Severity of Symptoms 3 Months After Infection with SARS-CoV-2 Compared to Test-Negative and Population Controls in the Netherlands. J. Infect. Dis..

[102] Tran V.-T., Porcher R., Pane I., Ravaud P. (2022). Course of post COVID-19 disease symptoms over time in the ComPaRe long COVID prospective e-cohort. Nat. Commun..

[103] Moghimi N., Di Napoli M., Biller J., Siegler J.E., Shekhar R., McCullough L.D., Harkins M.S., Hong E., Alaouieh D.A., Mansueto G. (2021). The Neurological Manifestations of Post-Acute Sequelae of SARS-CoV-2 Infection. Curr. Neurol. Neurosci. Rep..

[104] Huang C., Huang L., Wang Y., Li X., Ren L., Gu X., Kang L., Guo L., Liu M., Zhou X. (2021). 6-month consequences of COVID-19 in patients discharged from hospital: A cohort study. Lancet.

[105] O’Mahoney L.L., Routen A., Gillies C., Ekezie W., Welford A., Zhang A., Karamchandani U., Simms-Williams N., Cassambai S., Ardavani A. (2022). The prevalence and long-term health effects of Long Covid among hospitalised and non-hospitalised populations: A systematic review and meta-analysis. eClinicalMedicine.

[106] Westman G., Zelano J. (2022). Epilepsy diagnosis after COVID-19: A population-wide study. Seizure.

[107] Massey D., Sawano M., Baker A.D., Güthe D.B., Güthe N., Shidlovsky S.P., Fisher L., Grady C.B., Caraballo C., Zhou T. (2023). Characterisation of internal tremors and vibration symptoms. BMJ Open.

[108] Otani K., Fukushima H., Matsuishi K. (2023). COVID-19 delirium and encephalopathy: Pathophysiology assumed in the first 3 years of the ongoing pandemic. Brain Disord..

[109] Xie Y., Xu E., Bowe B., Al-Aly Z. (2022). Long-term cardiovascular outcomes of COVID-19. Nat. Med..

[110] Bereda G. (2023). How is ischemic stroke linked to a COVID-19-infected patient different from other cardiovascular risk factors? a case report. Ann. Med. Surg..

[111] Tonkal A., Alamri A.A., Al Maghrabi S.J., Mozahim N.F., Mozahim S.F., Alsubaie S.A., Alsehly A.A., Alshuaibi R.O., Alotaibi L.A., Qashgari F.S. (2022). Cranial Nerve Impairment Associated With COVID-19 Infections: A Systematic Review. Cureus.

[112] Heidari M.E., Nazemi P., Feizabad E., Beiranvand F., Afzali M. (2023). Cranial nerve involvement among COVID-19 survivors. Front. Neurol..

[113] Miller M.A. (2015). The role of sleep and sleep disorders in the development, diagnosis, and management of neurocognitive disorders. Front. Neurol..

[114] Marchi M., Grenzi P., Serafini V., Capoccia F., Rossi F., Marrino P., Pingani L., Galeazzi G.M., Ferrari S. (2023). Psychiatric symptoms in Long-COVID patients: A systematic review. Front. Psychiatry.

[115] Xu E., Xie Y., Al-Aly Z. (2022). Long-term neurologic outcomes of COVID-19. Nat. Med..

[116] Zawilska J.B., Kuczyńska K. (2022). Psychiatric and neurological complications of long COVID. J. Psychiatr. Res..

[117] Giraldo G.S.P., Ali S.T., Kang A.K., Patel T.R., Budhiraja S., Gaelen J.I., Lank G.K., Clark J.R., Mukherjee S., Singer T. (2023). Neurologic Manifestations of Long COVID Differ Based on Acute COVID-19 Severity. Ann. Neurol..

[118] Orrù G., Bertelloni D., Diolaiuti F., Mucci F., Di Giuseppe M., Biella M., Gemignani A., Ciacchini R., Conversano C. (2021). Long-COVID Syndrome? A Study on the Persistence of Neurological, Psychological and Physiological Symptoms. Healthcare.

[119] Williams S., Wynford-Thomas R., Robertson N.P. (2021). Long-COVID: Neurological manifestations and management. J. Neurol..

[120] Pilotto A., Cristillo V., Piccinelli S.C., Zoppi N., Bonzi G., Sattin D., Schiavolin S., Raggi A., Canale A., Gipponi S. (2021). Long-term neurological manifestations of COVID-19: Prevalence and predictive factors. Neurol. Sci..

[121] Li X., Hu H., Cheng Y. (2023). Neurological manifestations of long COVID: A single-center one-year experience [Letter]. Neuropsychiatr. Dis. Treat..

[122] Soares M.N., Eggelbusch M., Naddaf E., Gerrits K., van der Schaaf M., van den Borst B., Wiersinga W.J., van Vugt M., Weijs P., Murray A.J. (2022). Skeletal muscle alterations in patients with acute Covid-19 and post-acute sequelae of Covid-19. J. Cachexia Sarcopenia Muscle.

[123] Montes-Ibarra M., Oliveira C.L., Orsso C.E., Landi F., Marzetti E., Prado C.M. (2022). The Impact of Long COVID-19 on Muscle Health. Clin. Geriatr. Med..

[124] Carmona-Torre F., Mínguez-Olaondo A., López-Bravo A., Tijero B., Grozeva V., Walcker M., Azkune-Galparsoro H., de Munain A.L., Alcaide A.B., Quiroga J. (2022). Dysautonomia in COVID-19 Patients: A Narrative Review on Clinical Course, Diagnostic and Therapeutic Strategies. Front. Neurol..

[125] Sauer M.C., Barlow P.B., Comellas A.P., Garg A. (2024). Anxiety and depression symptoms among patients with long COVID: A retrospective cohort study. Eur. Arch. Psychiatry Clin. Neurosci..

[126] Perna G., Caldirola D. (2020). COVID-19 and panic disorder: Clinical considerations for the most physical of mental disorders. Rev. Bras. Psiquiatr..

[127] Mazza M.G., De Lorenzo R., Conte C., Poletti S., Vai B., Bollettini I., Melloni E.M.T., Furlan R., Ciceri F., Rovere-Querini P. (2020). Anxiety and depression in COVID-19 survivors: Role of inflammatory and clinical predictors. Brain Behav. Immun..

[128] Benke C., Wallenfels L.-M., Bleichhardt G.M., Melzig C.A. (2024). Health anxiety amplifies fearful responses to illness-related imagery. Sci. Rep..

[129] A Shetty P., Ayari L., Madry J., Betts C., Robinson D.M., Kirmani B.F. (2023). The Relationship Between COVID-19 and the Development of Depression: Implications on Mental Health. Neurosci. Insights.

[130] Deng J., Zhou F., Hou W., Silver Z., Wong C.Y., Chang O., Huang E., Zuo Q.K. (2021). The prevalence of depression, anxiety, and sleep disturbances in COVID-19 patients: A meta-analysis. Ann. N. Y. Acad. Sci..

[131] Lopes L.d.S., Silva R.O., Lima G.d.S., Costa A.C.d.A., Barros D.F., Silva-Néto R.P. (2021). Is there a common pathophysiological mechanism between COVID-19 and depression?. Acta Neurol. Belg..

[132] Rogers J.P., Chesney E., Oliver D., Pollak T.A., McGuire P., Fusar-Poli P., Zandi M.S., Lewis G., David A.S. (2020). Psychiatric and neuropsychiatric presentations associated with severe coronavirus infections: A systematic review and meta-analysis with comparison to the COVID-19 pandemic. Lancet Psychiatry.

[133] E Sommer I., Bakker P.R. (2020). What can psychiatrists learn from SARS and MERS outbreaks?. Lancet Psychiatry.

[134] Salari N., Hosseinian-Far A., Jalali R., Vaisi-Raygani A., Rasoulpoor S., Mohammadi M., Rasoulpoor S., Khaledi-Paveh B. (2020). Prevalence of stress, anxiety, depression among the general population during the COVID-19 pandemic: A systematic review and meta-analysis. Glob. Health.

[135] Zhao S., Martin E.M., Reuken P.A., Scholcz A., Ganse-Dumrath A., Srowig A., Utech I., Kozik V., Radscheidt M., Brodoehl S. (2024). Long COVID is associated with severe cognitive slowing: A multicentre cross-sectional study. eClinicalMedicine.

[136] Fernández-De-Las-Peñas C., Martín-Guerrero J.D., Cancela-Cilleruelo I., Moro-López-Menchero P., Rodríguez-Jiménez J., Pellicer-Valero O.J. (2023). Trajectory curves of post-COVID anxiety/depressive symptoms and sleep quality in previously hospitalized COVID-19 survivors: The LONG-COVID-EXP-CM multicenter study. Psychol. Med..

[137] Gross M., Lansang N.M., Gopaul U., Ogawa E.F., Heyn P.C., Santos F.H., Sood P., Zanwar P.P., Schwertfeger J., Faieta J. (2023). What Do I Need to Know About Long-Covid-related Fatigue, Brain Fog, and Mental Health Changes?. Arch. Phys. Med. Rehabil..

[138] Van Ameringen M., Patterson B., Turna J., Lethbridge G., Bergmann C.G., Lamberti N., Rahat M., Sideris B., Francisco A., Fineberg N. (2022). Obsessive-compulsive disorder during the COVID-19 pandemic. J. Psychiatr. Res..

[139] Demaria F., Pontillo M., Di Vincenzo C., Di Luzio M., Vicari S. (2022). Hand Washing: When Ritual Behavior Protects! Obsessive–Compulsive Symptoms in Young People during the COVID-19 Pandemic: A Narrative Review. J. Clin. Med..

[140] Samper-Pardo M., Oliván-Blázquez B., Magallón-Botaya R., Méndez-López F., Bartolomé-Moreno C., León-Herrera S. (2023). The emotional well-being of Long COVID patients in relation to their symptoms, social support and stigmatization in social and health services: A qualitative study. BMC Psychiatry.

[141] Iovino P., Vellone E., Cedrone N., Riegel B. (2023). A Middle-Range Theory of Social Isolation in Chronic Illness. Int. J. Environ. Res. Public Health.

[142] Kingstone T., Taylor A.K., O’Donnell C.A., Atherton H., Blane D.N., Chew-Graham C.A. (2020). Finding the ‘right’ GP: A qualitative study of the experiences of people with long-COVID. BJGP Open.

[143] Kubota T., Kuroda N., Sone D. (2023). Neuropsychiatric aspects of long COVID: A comprehensive review. Psychiatry Clin. Neurosci..

[144] Badenoch J.B., Rengasamy E.R., Watson C., Jansen K., Chakraborty S., Sundaram R.D., Hafeez D., Burchill E., Saini A., Thomas L. (2022). Persistent neuropsychiatric symptoms after COVID-19: A systematic review and meta-analysis. Brain Commun..

[145] Whiteside D.M., Basso M.R., Naini S.M., Porter J., Holker E., Waldron E.J., Melnik T.E., Niskanen N., Taylor S.E. (2022). Outcomes in post-acute sequelae of COVID-19 (PASC) at 6 months post-infection Part 1: Cognitive functioning. Clin. Neuropsychol..

[146] Krishnan K., Miller A.K., Reiter K., Bonner-Jackson A. (2022). Neurocognitive Profiles in Patients With Persisting Cognitive Symptoms Associated With COVID-19. Arch. Clin. Neuropsychol..

[147] Aiello E.N., Fiabane E., Manera M.R., Radici A., Grossi F., Ottonello M., Pain D., Pistarini C. (2022). Screening for cognitive sequelae of SARS-CoV-2 infection: A comparison between the Mini-Mental State Examination (MMSE) and the Montreal Cognitive Assessment (MoCA). Neurol. Sci..

[148] Dressing A., Bormann T., Blazhenets G., Schroeter N., Walter L.I., Thurow J., August D., Hilger H., Stete K., Gerstacker K. (2022). Neuropsychologic Profiles and Cerebral Glucose Metabolism in Neurocognitive Long COVID Syndrome. J. Nucl. Med..

[149] Ferrando S.J., Dornbush R., Lynch S., Shahar S., Klepacz L., Karmen C.L., Chen D., Lobo S.A., Lerman D. (2022). Neuropsychological, Medical, and Psychiatric Findings After Recovery from Acute COVID-19: A Cross-sectional Study. Psychosom.

[150] Crivelli L., Palmer K., Calandri I., Guekht A., Beghi E., Carroll W., Frontera J., García-Azorín D., Westenberg E., Winkler A.S. (2022). Changes in cognitive functioning after COVID-19: A systematic review and meta-analysis. Alzheimer’s Dement..

[151] García-Sánchez C., Calabria M., Grunden N., Pons C., Arroyo J.A., Gómez-Anson B., Lleó A., Alcolea D., Belvís R., Morollón N. (2022). Neuropsychological deficits in patients with cognitive complaints after COVID-19. Brain Behav..

[152] Giurgi-Oncu C., Tudoran C., Pop G.N., Bredicean C., Pescariu S.A., Giurgiuca A., Tudoran M. (2021). Cardiovascular Abnormalities and Mental Health Difficulties Result in a Reduced Quality of Life in the Post-Acute COVID-19 Syndrome. Brain Sci..

[153] Houben-Wilke S., Goërtz Y.M., Delbressine J.M., Vaes A.W., Meys R., Machado F.V., van Herck M., Burtin C., Posthuma R., Franssen F.M. (2022). The Impact of Long COVID-19 on Mental Health: Observational 6-Month Follow-Up Study. JMIR Ment. Health.

[154] Benítez I.D.M., Moncusí-Moix A.M., Vaca R.M., Gort-Paniello C.M., Minguez O.M., Santisteve S.M., Carmona P.M., Torres G., Fagotti J., Labarca G. (2022). Sleep and Circadian Health of Critical COVID-19 Survivors 3 Months After Hospital Discharge. Crit. Care Med..

[155] Ferrucci R., Dini M., Rosci C., Capozza A., Groppo E., Reitano M.R., Allocco E., Poletti B., Brugnera A., Bai F. (2022). One-year cognitive follow-up of COVID-19 hospitalized patients. Eur. J. Neurol..

[156] Sujan S.H., Tasnim R., Haghighathoseini A., Hasan M.M., Islam S. (2023). Investigating posttraumatic stress disorder among COVID-19 recovered patients: A cross-sectional study. Heliyon.

[157] Shiwaku H., Doi S., Miyajima M., Matsumoto Y., Fujino J., Hirai N., Jitoku D., Takagi S., Tamura T., Maruo T. (2021). Novel brief screening scale, Tokyo Metropolitan Distress Scale for Pandemic (TMDP), for assessing mental and social stress of medical personnel in COVID-19 pandemic. Psychiatry Clin. Neurosci..

[158] George K.M., Lutsey P.L., Selvin E., Palta P., Windham B.G., Folsom A.R. (2019). Association Between Thyroid Dysfunction and Incident Dementia in the Atherosclerosis Risk in Communities Neurocognitive Study. J. Endocrinol. Metab..

[159] Obeid R., Andrès E., Češka R., Hooshmand B., Guéant-Rodriguez R.-M., Prada G.I., Sławek J., Traykov L., Van B.T., Várkonyi T. (2024). Diagnosis, Treatment and Long-Term Management of Vitamin B12 Deficiency in Adults: A Delphi Expert Consensus. J. Clin. Med..

[160] Boyarchuk O., Volianska L. (2024). Autoimmunity and long COVID in children. Rheumatology.

[161] Graham E.L., Koralnik I.J., Liotta E.M. (2022). Therapeutic Approaches to the Neurologic Manifestations of COVID-19. Neurotherapeutics.

[162] Dennis A., Cuthbertson D.J., Wootton D., Crooks M., Gabbay M., Eichert N., Mouchti S., Pansini M., Roca-Fernandez A., Thomaides-Brears H. (2023). Multi-organ impairment and long COVID: A 1-year prospective, longitudinal cohort study. J. Royal Soc. Med..

[163] Lin L., Al-Faraj A., Ayub N., Bravo P., Das S., Ferlini L., Karakis I., Lee J.W., Mukerji S.S., Newey C.R. (2021). Electroencephalographic Abnormalities are Common in COVID-19 and are Associated with Outcomes. Ann. Neurol..

[164] Komaroff A.L., Lipkin W.I. (2023). ME/CFS and Long COVID share similar symptoms and biological abnormalities: Road map to the literature. Front. Med..

[165] Reiss A.B., Greene C., Dayaramani C., Rauchman S.H., Stecker M.M., De Leon J., Pinkhasov A. (2023). Long COVID, the Brain, Nerves, and Cognitive Function. Neurol. Int..

[166] Kuut T.A., Müller F., Csorba I., Braamse A., Aldenkamp A., Appelman B., Assmann-Schuilwerve E., Geerlings S.E., Gibney K.B., Kanaan R.A.A. (2023). Efficacy of Cognitive-Behavioral Therapy Targeting Severe Fatigue Following Coronavirus Disease 2019: Results of a Randomized Controlled Trial. Clin. Infect. Dis..

[167] Kutashov V.A. (2021). Actovegin use in patients with cognitive impairment after coronavirus infection (COVID-19). Neurol. Neuropsychiatry Psychosom..

[168] Fu Y., Song Y., Li Y., Sanchez-Vidana D.I., Zhang J.J., Lau W.K., Tan D.G.H., Ngai S.P.C., Lau B.W.-M. (2024). The effect of mindfulness meditation on depressive symptoms during the COVID-19 pandemic: A systematic review and meta-analysis. Sci. Rep..

[169] Behl T., Rocchetti G., Chadha S., Zengin G., Bungau S., Kumar A., Mehta V., Uddin S., Khullar G., Setia D. (2021). Phytochemicals from Plant Foods as Potential Source of Antiviral Agents: An Overview. Pharmaceuticals.

[170] Colas C., Le Berre Y., Fanget M., Savall A., Killian M., Goujon I., Labeix P., Bayle M., Féasson L., Roche F. (2023). Physical Activity in Long COVID: A Comparative Study of Exercise Rehabilitation Benefits in Patients with Long COVID, Coronary Artery Disease and Fibromyalgia. Int. J. Environ. Res. Public Health.

[171] Zeraatkar D., Ling M., Kirsh S., Jassal T., Shahab M., Movahed H., Talukdar J.R., Walch A., Chakraborty S., Turner T. (2024). Interventions for the management of long covid (post-covid condition): Living systematic review. BMJ.

[172] Mazza M.G., Zanardi R., Palladini M., Rovere-Querini P., Benedetti F. (2022). Rapid response to selective serotonin reuptake inhibitors in post-COVID depression. Eur. Neuropsychopharmacol..

[173] Rus C.P., de Vries B.E.K., de Vries I.E.J., Nutma I., Kooij J.J.S. (2023). Treatment of 95 post-Covid patients with SSRIs. Sci. Rep..

[174] Butzin-Dozier Z., Ji Y., Deshpande S., Hurwitz E., Anzalone A.J., Coyle J., Shi J., Mertens A., van der Laan M.J., Colford J.M. (2024). SSRI use during acute COVID-19 and risk of Long COVID among patients with depression. BMC Med..

[175] Pashaei Y. (2021). Drug repurposing of selective serotonin reuptake inhibitors: Could these drugs help fight COVID-19 and save lives?. J. Clin. Neurosci..

[176] Sanilevici M., Reuveni O., Lev-Ari S., Golland Y., Levit-Binnun N. (2021). Mindfulness-Based Stress Reduction Increases Mental Wellbeing and Emotion Regulation During the First Wave of the COVID-19 Pandemic: A Synchronous Online Intervention Study. Front. Psychol..

[177] Guezguez F., Romdhani M., Boutaleb-Joutei A., Chamari K., Ben Saad H. (2023). Management of long-COVID-19 patients with sleep disorders: Practical advice to general practitioners. Libyan J. Med..

[178] Anderson G.M. (2021). Fluvoxamine, melatonin and COVID-19. Psychopharmacol.

[179] Anderson G., Reiter R.J. (2020). Melatonin: Roles in influenza, Covid-19, and other viral infections. Rev. Med. Virol..

[180] Weise A., Ott E., Hersche R. (2024). Energy Management Education in Persons with Long COVID-Related Fatigue: Insights from Focus Group Results on Occupational Therapy Approach. Healthcare.

[181] Spencer L.H., Hendry A., Makanjuola A., Anthony B.F., Davies J., Pisavadia K., Hughes D., Fitzsimmons D., Wilkinson C., Edwards R.T. (2023). What interventions or best practice are there to support people with Long COVID, or similar post-viral conditions or conditions characterised by fatigue, to return to normal activities: A rapid review. medRxiv.

[182] Sanal-Hayes N.E.M., Mclaughlin M., Hayes L.D., Mair J.L., Ormerod J., Carless D., Hilliard N., Meach R., Ingram J., Sculthorpe N.F. (2023). A scoping review of ‘Pacing’ for management of Myalgic Encephalomyelitis/Chronic Fatigue Syndrome (ME/CFS): Lessons learned for the long COVID pandemic. J. Transl. Med..

[183] Younes S. (2024). The role of nutrition on the treatment of COVID-19. Hum. Nutr. Metab..

[184] Fernández-De-Las-Peñas C., Nijs J., Neblett R., Polli A., Moens M., Goudman L., Patil M.S., Knaggs R.D., Pickering G., Arendt-Nielsen L. (2022). Phenotyping Post-COVID Pain as a Nociceptive, Neuropathic, or Nociplastic Pain Condition. Biomedicines.

[185] Di Stefano G., Falco P., Galosi E., Di Pietro G., Leone C., Truini A. (2023). A systematic review and meta-analysis of neuropathic pain associated with coronavirus disease2019. Eur. J. Pain..

[186] El-Tallawy S.N., Perglozzi J.V., Ahmed R.S., Kaki A.M., Nagiub M.S., LeQuang J.K., Hadarah M.M. (2023). Pain Management in the Post-COVID Era—An Update: A Narrative Review. Pain. Ther..

[187] Taube M. (2023). Depression and brain fog as long-COVID mental health consequences: Difficult, complex and partially successful treatment of a 72-year-old patient—A case report. Front. Psychiatry.

[188] Veronese N., Bonica R., Cotugno S., Tulone O., Camporeale M., Smith L., Trott M., Bruyere O., Mirarchi L., Rizzo G. (2022). Interventions for Improving Long COVID-19 Symptomatology: A Systematic Review. Viruses.

[189] Samper-Pardo M., León-Herrera S., Oliván-Blázquez B., Méndez-López F., Domínguez-García M., Sánchez-Recio R. (2023). Effectiveness of a telerehabilitation intervention using ReCOVery APP of long COVID patients: A randomized, 3-month follow-up clinical trial. Sci. Rep..

[190] Kapur A. (2020). Is Methylphenidate Beneficial and Safe in Pharmacological Cognitive Enhancement?. CNS Drugs.

[191] Clark P., Rosenberg P., Oh E.S., Parker A., Vannorsdall T., Azola A., Nickles E., Galiatsatos P., Malik M. (2024). Methylphenidate for the Treatment of Post-COVID Cognitive Dysfunction (Brain Fog). J. Med. Cases.

[192] Carrasco-Garrido P., Fernández-De-Las-Peñas C., Hernández-Barrera V., Palacios-Ceña D., Jiménez-Trujillo I., Gallardo-Pino C. (2022). Benzodiazepines and Z-hypnotics consumption in long-COVID-19 patients: Gender differences and associated factors. Front. Med..

[193] Lenze E.J., Reiersen A.M., Zorumski C.F., Santosh P.J. (2023). Beyond “Psychotropic”. J. Clin. Psychiatry.

[194] Lu Y., Ho C.S., Liu X., Chua A.N., Wang W., McIntyre R.S., Ho R.C. (2017). Chronic administration of fluoxetine and pro-inflammatory cytokine change in a rat model of depression. PLoS ONE.

[195] Carpinteiro A., Edwards M.J., Hoffmann M., Kochs G., Gripp B., Weigang S., Adams C., Carpinteiro E., Gulbins A., Keitsch S. (2020). Pharmacological Inhibition of Acid Sphingomyelinase Prevents Uptake of SARS-CoV-2 by Epithelial Cells. Cell Rep. Med..

[196] Kornhuber J., Muehlbacher M., Trapp S., Pechmann S., Friedl A., Reichel M., Mühle C., Terfloth L., Groemer T.W., Spitzer G.M. (2011). Identification of Novel Functional Inhibitors of Acid Sphingomyelinase. PLoS ONE.

[197] Homolak J., Kodvanj I. (2020). Widely available lysosome targeting agents should be considered as potential therapy for COVID-19. Int. J. Antimicrob. Agents.

[198] Schlienger R.G., Meier C.R. (2003). Effect of Selective Serotonin Reuptake Inhibitors on Platelet Activation. Am. J. Cardiovasc. Drugs.

[199] Reiersen A.M., Vayttaden S.J., Sukhatme V.V. (2021). Fluvoxamine: A Review of Its Mechanism of Action and Its Role in COVID-19. Front. Pharmacol..

[200] Russo S., Fiani F., Napoli C. (2024). Remote Eye Movement Desensitization and Reprocessing Treatment of Long-COVID-19 and Post-COVID-Related Traumatic Disorders: An Innovative Approach. Brain Sci..

[201] Fan Y., Shi Y., Zhang J., Sun D., Wang X., Fu G., Mo D., Wen J., Xiao X., Kong L. (2021). The effects of narrative exposure therapy on COVID-19 patients with post-traumatic stress symptoms: A randomized controlled trial. J. Affect. Disord..

[202] Walker J., Muench A., Perlis M., Vargas I. (2022). Cognitive Behavioral Therapy for Insomnia (CBT-I): A Primer. Clin. Psychol. Spéc. Educ..

[203] Becker P.M. (2021). Overview of sleep management during COVID-19. Sleep Med..

[204] Soyka M., Wild I., Caulet B., Leontiou C., Lugoboni F., Hajak G. (2023). Long-term use of benzodiazepines in chronic insomnia: A European perspective. Front. Psychiatry.

[205] Barrea L., Verde L., Grant W.B., Frias-Toral E., Sarno G., Vetrani C., Ceriani F., Garcia-Velasquez E., Contreras-Briceño J., Savastano S. (2022). Vitamin D: A Role Also in Long COVID-19?. Nutrients.

[206] Mantle D., Hargreaves I.P., Domingo J.C., Castro-Marrero J. (2024). Mitochondrial Dysfunction and Coenzyme Q10 Supplementation in Post-Viral Fatigue Syndrome: An Overview. Int. J. Mol. Sci..

[207] Castro-Marrero J., Segundo M.J., Lacasa M., Martinez-Martinez A., Sentañes R.S., Alegre-Martin J. (2021). Effect of Dietary Coenzyme Q10 Plus NADH Supplementation on Fatigue Perception and Health-Related Quality of Life in Individuals with Myalgic Encephalomyelitis/Chronic Fatigue Syndrome: A Prospective, Randomized, Double-Blind, Placebo-Controlled Trial. Nutrients.

[208] Xie Y., Choi T., Al-Aly Z. (2023). Association of Treatment with Nirmatrelvir and the Risk of Post–COVID-19 Condition. JAMA Intern. Med..

[209] Antar A.A.R., Peluso M.J. (2025). CROI 2023: Acute and Post-Acute COVID-19. Top. Antivir. Med..

[210] Cohen A.K., Jaudon T.W., Schurman E.M., Kava L., Vogel J.M., Haas-Godsil J., Lewis D., Crausman S., Leslie K., Bligh S.C. (2023). Impact of extended-course oral nirmatrelvir/ritonavir (Paxlovid) in established Long COVID: Case series and research considerations. Res. Sq..

[211] Fernández-De-Las-Peñas C., Torres-Macho J., Macasaet R., Velasco J.V., Ver A.T., Carandang T.H.D.C., Guerrero J.J., Franco-Moreno A., Chung W., Notarte K.I. (2024). Presence of SARS-CoV-2 RNA in COVID-19 survivors with post-COVID symptoms: A systematic review of the literature. Clin. Chem. Lab. Med..

[212] Sharif-Askari F.S., Alsayed H.A.H., Sharif-Askari N.S., Hussain A.A.S., Al-Muhsen S., Halwani R. (2024). Nirmatrelvir plus ritonavir reduces COVID-19 hospitalization and prevents long COVID in adult outpatients. Sci. Rep..

[213] Congdon S., Narrowe Z., Yone N., Gunn J., Deng Y., Nori P., Cowman K., Islam M., Rikin S., Starrels J. (2023). Nirmatrelvir/ritonavir and risk of long COVID symptoms: A retrospective cohort study. Sci. Rep..

[214] Fernández-De-Las-Peñas C., Torres-Macho J., Catahay J.A., Macasaet R., Velasco J.V., Macapagal S., Caldararo M., Henry B.M., Lippi G., Franco-Moreno A. (2024). Is antiviral treatment at the acute phase of COVID-19 effective for decreasing the risk of long-COVID? A systematic review. Infection.

[215] Al-Aly Z. (2023). Prevention of long COVID: Progress and challenges. Lancet Infect. Dis..

[216] Sam K.S., Khosla P., Taneja V., Dessai R. (2024). Casirivimab-imdevimab monoclonal antibody treatment for an immunocompromised patient with persistent SARS-CoV-2 infection: A case report. Commun. Med..

[217] Negrut N., Codrean A., Hodisan I., Bungau S., Tit D.M., Marin R., Behl T., Banica F., Diaconu C.C., Nistor-Cseppento D.C. (2021). Efficiency of antiviral treatment in COVID-19. Exp. Ther. Med..

[218] Gaylis N.B., Ritter A., Kelly S.A., Pourhassan N.Z., Tiwary M., Sacha J.B., Hansen S.G., Recknor C., Yang O.O. (2022). Reduced Cell Surface Levels of C-C Chemokine Receptor 5 and Immunosuppression in Long Coronavirus Disease 2019 Syndrome. Clin. Infect. Dis..

[219] Sizyakina L., Zakurskaya V., Guryanova S. (2023). Glucosaminylmuramyl dipeptide efficacy in post-COVID-19 patient rehabilitation treatment. Infect. Dis. News Opin. Train..

[220] McIntyre R.S., Phan L., Kwan A.T.H., Mansur R.B., Rosenblat J.D., Guo Z., Le G.H., Lui L.M.W., Teopiz K.M., Ceban F. (2024). Vortioxetine for the treatment of post-COVID-19 condition: A randomized controlled trial. Brain.

[221] Lau R.I., Su Q., Lau I.S.F., Ching J.Y.L., Wong M.C.S., Lau L.H.S., Tun H.M., Mok C.K.P., Chau S.W.H., Tse Y.K. (2024). A synbiotic preparation (SIM01) for post-acute COVID-19 syndrome in Hong Kong (RECOVERY): A randomised, double-blind, placebo-controlled trial. Lancet Infect. Dis..

[222] Liu Q., Mak J.W.Y., Su Q., Yeoh Y.K., Lui G.C.-Y., Ng S.S.S., Zhang F., Li A.Y.L., Lu W., Hui D.S.-C. (2022). Gut microbiota dynamics in a prospective cohort of patients with post-acute COVID-19 syndrome. Gut.

[223] Zilberman-Itskovich S., Catalogna M., Sasson E., Elman-Shina K., Hadanny A., Lang E., Finci S., Polak N., Fishlev G., Korin C. (2022). Hyperbaric oxygen therapy improves neurocognitive functions and symptoms of post-COVID condition: Randomized controlled trial. Sci. Rep..

[224] Santana K., França E., Sato J., Silva A., Queiroz M., de Farias J., Rodrigues D., Souza I., Ribeiro V., Caparelli-Dáquer E. (2023). Non-invasive brain stimulation for fatigue in post-acute sequelae of SARS-CoV-2 (PASC). Brain Stimul..

[225] Elbannaa R., Mogahed H., Zahran M., Mohamed E. (2022). The effect of photobiomodulation versus placebo on functional capacity and fatigability in post COVID-19 elderly. Adv. Rehabil..

[226] Florencio L.L., Fernández-De-Las-Peñas C. (2022). Long COVID: Systemic inflammation and obesity as therapeutic targets. Lancet Respir. Med..

[227] Palaiodimou L., Stefanou M., Katsanos A.H., Fragkou P.C., Papadopoulou M., Moschovos C., Michopoulos I., Kokotis P., Bakirtzis C., Naska A. (2021). Prevalence, clinical characteristics and outcomes of Guillain−Barré syndrome spectrum associated with COVID-19: A systematic review and meta-analysis. Eur. J. Neurol..

[228] Oaklander A.L., Mills A.J., Kelley M., Toran L.S., Smith B., Dalakas M.C., Nath A. (2022). Peripheral Neuropathy Evaluations of Patients With Prolonged Long COVID. Neurol. Neuroimmunol. Neuroinflammation.

[229] Yang C.-P., Chang C.-M., Yang C.-C., Pariante C.M., Su K.-P. (2022). Long COVID and long chain fatty acids (LCFAs): Psychoneuroimmunity implication of omega-3 LCFAs in delayed consequences of COVID-19. Brain Behav. Immun..

[230] Chang J.P.-C., Pariante C.M., Su K.-P. (2020). Omega-3 fatty acids in the psychological and physiological resilience against COVID-19. Prostaglandins Leukot. Essent. Fat. Acids.

[231] Liu T.-H., Ho C.-H., Chen D.T.-L., Wu J.-Y., Huang P.-Y., Lai C.-C., Hsieh K.-Y., Su K.-P. (2023). Omega-3 polyunsaturated fatty acids and the psychiatric post-acute sequelae of COVID-19: A one-year retrospective cohort analysis of 33,908 patients. Brain Behav. Immun..

[232] Rodríguez-Vera D., Salazar J.R., Soriano-Ursúa M.A., Guzmán-Pérez J., Vergara-Castañeda A., Muñoz-Durán H., Ramírez-Velez G.L., Vivar-Sierra A., Naranjo-Navarro C.R., Meza-Meneses P.A. (2024). Effectiveness of Omega-3 Fatty Acid Supplementation in Improving the Metabolic and Inflammatory Profiles of Mexican Adults Hospitalized with COVID-19. Diseases.

[233] Sarkar A., Speiser E., Dara S., Ogedegbe C., Chinnery P., Estanbouli M.-T., Kasselman L., Kligler B., Paleoudis E.G., Parulekar M. (2024). The Feasibility of Omega-3 Supplementation Compared to Placebo in the Management of Long COVID Symptoms Among Healthcare Workers: A Randomized Controlled Trial. Cureus.

[234] Líška D., Liptaková E., Babičová A., Batalik L., Baňárová P.S., Dobrodenková S. (2022). What is the quality of life in patients with long COVID compared to a healthy control group?. Front. Public Health.

[235] Malesevic S., Sievi N.A., Baumgartner P., Roser K., Sommer G., Schmidt D., Vallelian F., Jelcic I., Clarenbach C.F., Kohler M. (2023). Impaired health-related quality of life in long-COVID syndrome after mild to moderate COVID-19. Sci. Rep..

[236] Sun C., Liu Z., Li S., Wang Y., Liu G. (2024). Impact of Long COVID on Health-Related Quality of Life Among Patients After Acute COVID-19 Infection: A Cross-Sectional Study. Inq. J. Health Care Organ. Provis. Financ..

[237] Romero-Rodríguez E., Perula-De-Torres L.Á., González-Lama J., Castro-Jiménez R.Á., Jiménez-García C., Priego-Pérez C., Vélez-Santamaría R., Simón-Vicente L., González-Santos J., González-Bernal J.J. (2023). Long COVID Symptomatology and Associated Factors in Primary Care Patients: The EPICOVID-AP21 Study. Healthcare.

[238] Vélez-Santamaría R., Fernández-Solana J., Méndez-López F., Domínguez-García M., González-Bernal J.J., Magallón-Botaya R., Oliván-Blázquez B., González-Santos J., Santamaría-Peláez M. (2023). Functionality, physical activity, fatigue and quality of life in patients with acute COVID-19 and Long COVID infection. Sci. Rep..

[239] Miskowiak K., Pedersen J., Gunnarsson D., Roikjer T., Podlekareva D., Hansen H., Dall C., Johnsen S. (2023). Cognitive impairments among patients in a long-COVID clinic: Prevalence, pattern and relation to illness severity, work function and quality of life. J. Affect. Disord..

[240] Li Z., Zhang Z., Zhang Z., Wang Z., Li H. (2023). Cognitive impairment after long COVID-19: Current evidence and perspectives. Front. Neurol..

[241] Liang L., Hou W., Li T., Liu H., Goodwin R., Lee T. (2023). Latent Profiles and Transitions of Daily Routine Disruptions Are Associated with Severity of Symptoms of Anxiety and Depression. Leis. Sci..

[242] Ladds E., Rushforth A., Wieringa S., Taylor S., Rayner C., Husain L., Greenhalgh T. (2020). Persistent symptoms after COVID-19: Qualitative study of 114 “long Covid” patients and draft quality principles for services. BMC Health Serv. Res..

[243] Parkin A., Davison J., Tarrant R., Ross D., Halpin S., Simms A., Salman R., Sivan M. (2021). A Multidisciplinary NHS COVID-19 Service to Manage Post-COVID-19 Syndrome in the Community. J. Prim. Care Community Health.

[244] Singh S., Roy D., Sinha K., Parveen S., Sharma G., Joshi G. (2020). Impact of COVID-19 and lockdown on mental health of children and adolescents: A narrative review with recommendations. Psychiatry Res..

[245] Sanchez-Gomez M., Giorgi G., Finstad G.L., Urbini F., Foti G., Mucci N., Zaffina S., León-Perez J.M. (2021). COVID-19 Pandemic as a Traumatic Event and Its Associations with Fear and Mental Health: A Cognitive-Activation Approach. Int. J. Environ. Res. Public Health.

[246] Bonati M., Campi R., Segre G. (2022). Psychological impact of the quarantine during the COVID-19 pandemic on the general European adult population: A systematic review of the evidence. Epidemiol. Psychiatr. Sci..

[247] Dos Santos E.R.R., de Paula J.L.S., Tardieux F.M., Costa-E-Silva V.N., Lal A., Leite A.F.B. (2021). Association between COVID-19 and anxiety during social isolation: A systematic review. World J. Clin. Cases.

[248] Pietrabissa G., Simpson S.G. (2020). Psychological Consequences of Social Isolation During COVID-19 Outbreak. Front. Psychol..

[249] Marashi M.Y., Nicholson E., Ogrodnik M., Fenesi B., Heisz J.J. (2021). A mental health paradox: Mental health was both a motivator and barrier to physical activity during the COVID-19 pandemic. PLoS ONE.

[250] Young E., Milligan K., Henze M., Johnson S., Weyman K. (2021). Caregiver burnout, gaps in care, and COVID-19. Can. Fam. Physician.

[251] Torres C., Maeda K., Johnson M., Jason L.A. (2024). Understanding Experiences of Youth with Long COVID: A Qualitative Approach. Children.

[252] Brackel C.L.H., Lap C.R., Buddingh E.P., van Houten M.A., van der Sande L.J.T.M., Langereis E.J., Bannier M.A.G.E., Pijnenburg M.W.H., Hashimoto S., Terheggen-Lagro S.W.J. (2021). Pediatric long-COVID: An overlooked phenomenon?. Pediatr. Pulmonol..

[253] Fink T.T., Marques H.H., Gualano B., Lindoso L., Bain V., Astley C., Martins F., Matheus D., Matsuo O.M., Suguita P. (2021). Persistent symptoms and decreased health-related quality of life after symptomatic pediatric COVID-19: A prospective study in a Latin American tertiary hospital. Clinics.

[254] Jason L.A., Johnson M., Torres C. (2023). Pediatric Post-Acute Sequelae of SARS-CoV-2 infection. Fatigue Biomed. Health Behav..

[255] Turkel S., Pao M. (2007). Late Consequences of Chronic Pediatric Illness. Psychiatr. Clin. N. Am..

[256] Hassan N.M., Salim H.S., Amaran S., Yunus N.I., Yusof N.A., Daud N., Fry D. (2023). Prevalence of mental health problems among children with long COVID: A systematic review and meta-analysis. PLoS ONE.

[257] Liu S.-T., Lin S.-C., Chang J.P.-C., Yang K.-J., Chu C.-S., Yang C.-C., Liang C.-S., Sun C.-F., Wang S.-C., Satyanarayanan S.K. (2023). The Clinical Observation of Inflammation Theory for Depression: The Initiative of the Formosa Long COVID Multicenter Study (FOCuS). Clin. Psychopharmacol. Neurosci..

[258] Byrne E.A. (2022). Understanding Long Covid: Nosology, social attitudes and stigma. Brain Behav. Immun..

[259] Shachar-Lavie I., Shorer M., Segal H., Fennig S., Ashkenazi-Hoffnung L. (2023). Mental health among children with long COVID during the COVID-19 pandemic. Eur. J. Pediatr..

[260] Ha E.K., Kim J.H., Han M.Y. (2023). Long COVID in children and adolescents: Prevalence, clinical manifestations, and management strategies. Clin. Exp. Pediatr..

[261] Borch L., Holm M., Knudsen M., Ellermann-Eriksen S., Hagstroem S. (2022). Long COVID symptoms and duration in SARS-CoV-2 positive children—A nationwide cohort study. Eur. J. Pediatr..

[262] Berg S.K., Nielsen S.D., Nygaard U., Bundgaard H., Palm P., Rotvig C., Christensen A.V. (2022). Long COVID symptoms in SARS-CoV-2-positive adolescents and matched controls (LongCOVIDKidsDK): A national, cross-sectional study. Lancet Child. Adolesc. Health.

[263] Tedjasukmana R., Budikayanti A., Islamiyah W.R., Witjaksono A.M.A.L., Hakim M. (2023). Sleep disturbance in post COVID-19 conditions: Prevalence and quality of life. Front. Neurol..

[264] Bothe K., Schabus M., Eigl E.-S., Kerbl R., Hoedlmoser K. (2022). Self-reported changes in sleep patterns and behavior in children and adolescents during COVID-19. Sci. Rep..

[265] Zavala M., Ireland G., Amin-Chowdhury Z., E Ramsay M., Ladhani S.N. (2022). Acute and Persistent Symptoms in Children With Polymerase Chain Reaction (PCR)–Confirmed Severe Acute Respiratory Syndrome Coronavirus 2 (SARS-CoV-2) Infection Compared With Test-Negative Children in England: Active, Prospective, National Surveillance. Clin. Infect. Dis..

[266] Swartz M.K. (2021). Post-COVID Conditions in Children. J. Pediatr. Health Care.

[267] Faux-Nightingale A., Saunders B., Burton C., Chew-Graham C.A., Somayajula G., Twohig H., Welsh V. (2024). Experiences and care needs of children with long COVID: A qualitative study. BJGP Open.

[268] Savino R., Polito A.N., Arcidiacono G., Poliseno M., Caputo S.L. (2022). Neuropsychiatric Disorders in Pediatric Long COVID-19: A Case Series. Brain Sci..

[269] Behnood S., Shafran R., Bennett S., Zhang A., O’Mahoney L., Stephenson T., Ladhani S., De Stavola B., Viner R., Swann O. (2022). Persistent symptoms following SARS-CoV-2 infection amongst children and young people: A meta-analysis of controlled and uncontrolled studies. J. Infect..

[270] Faux-Nightingale A., Burton C., Twohig H., Blagojevic-Bucknall M., Carroll W., A Chew-Graham C., Dunn K., Gilchrist F., Helliwell T., Lawton O. (2023). Symptom patterns and life with post-acute COVID-19 in children aged 8–17 years: A mixed-methods study protocol. BJGP Open.

[271] MacLean A., Wild C., Hunt K., Nettleton S., Skea Z.C., Ziebland S. (2023). Impact of Long Covid on the school experiences of children and young people: A qualitative study. BMJ Open.

[272] Ng C.S.M., Ng S.S.L. (2022). Impact of the COVID-19 pandemic on children’s mental health: A systematic review. Front. Psychiatry.

[273] Gonzalez-Aumatell A., Bovo M.V., Carreras-Abad C., Cuso-Perez S., Marsal È.D., Coll-Fernández R., Calvo A.G., Giralt-López M., Cantallops A.E., Moron-Lopez S. (2022). Social, Academic and Health Status Impact of Long COVID on Children and Young People: An Observational, Descriptive, and Longitudinal Cohort Study. Children.

[274] Pellegrino R., Chiappini E., Licari A., Galli L., Marseglia G.L. (2022). Prevalence and clinical presentation of long COVID in children: A systematic review. Eur. J. Pediatr..

[275] Stephenson T., Shafran R., Ladhani S.N. (2022). Long COVID in children and adolescents. Curr. Opin. Infect. Dis..

